# MR Intensity Normalization Methods Impact Sequence Specific Radiomics Prognostic Model Performance in Primary and Recurrent High-Grade Glioma

**DOI:** 10.3390/cancers15030965

**Published:** 2023-02-02

**Authors:** Patrick Salome, Francesco Sforazzini, Gianluca Grugnara, Andreas Kudak, Matthias Dostal, Christel Herold-Mende, Sabine Heiland, Jürgen Debus, Amir Abdollahi, Maximilian Knoll

**Affiliations:** 1 Clinical Cooperation Unit (CCU) Radiation Oncology, German Cancer Research Centre, INF 280, 69120 Heidelberg, Germany; 2 Heidelberg Medical Faculty, Heidelberg University, 69120 Heidelberg, Germany; 3 German Cancer Consortium (DKTK) Core Centre Heidelberg, 69120 Heidelberg, Germany; 4 Heidelberg Ion-Beam Therapy Centre (HIT), INF 450, 69120 Heidelberg, Germany; 5 Department of Neuroradiology, Heidelberg University Hospital, 69120 Heidelberg, Germany; 6 Department of Radiation Oncology, Heidelberg University Hospital, INF 400, 69120 Heidelberg, Germany; 7 CCU Radiation Therapy, German Cancer Research Centre, INF 280, 69120 Heidelberg, Germany; 8 Brain Tumour Group, European Organization for Research and Treatment of Cancer, 1200 Brussels, Belgium; 9 Division of Neurosurgical Research, Department of Neurosurgery, Heidelberg University Hospital, 69120 Heidelberg, Germany

**Keywords:** multiparametric MRI, image preprocessing, intensity harmonization, intensity standardization, high-grade glioma, radiomics signatures

## Abstract

**Simple Summary:**

As magnetic resonance (MR) intensities are acquired in arbitrary units, scans from different scanners are not directly comparable; thus, intensity normalization is essential. In this study, we assess the impact of normalization methods on prognostic radiomics models in primary and recurrent high-grade glioma on different MR sequences. Furthermore, we present a methodology that allows for the handling of radiomics performance discrepancy due to MR intensity normalization.

**Abstract:**

Purpose: This study investigates the impact of different intensity normalization (IN) methods on the overall survival (OS) radiomics models’ performance of MR sequences in primary (pHGG) and recurrent high-grade glioma (rHGG). Methods: MR scans acquired before radiotherapy were retrieved from two independent cohorts (rHGG C1: 197, pHGG C2: 141) from multiple scanners (15, 14). The sequences are T1 weighted (w), contrast-enhanced T1w (T1wce), T2w, and T2w-FLAIR. Sequence-specific significant features (SF) associated with OS, extracted from the tumour volume, were derived after applying 15 different IN methods. Survival analyses were conducted using Cox proportional hazard (CPH) and Poisson regression (POI) models. A ranking score was assigned based on the 10-fold cross-validated (CV) concordance index (C-I), mean square error (MSE), and the Akaike information criterion (AICs), to evaluate the methods’ performance. Results: Scatter plots of the 10-CV C-I and MSE against the AIC showed an impact on the survival predictions between the IN methods and MR sequences (C1/C2 C-I range: 0.62–0.71/0.61–0.72, MSE range: 0.20–0.42/0.13–0.22). White stripe showed stable results for T1wce (C1/C2 C-I: 0.71/0.65, MSE: 0.21/0.14). Combat (0.68/0.62, 0.22/0.15) and histogram matching (HM, 0.67/0.64, 0.22/0.15) showed consistent prediction results for T2w models. They were also the top-performing methods for T1w in C2 (Combat: 0.67, 0.13; HM: 0.67, 0.13); however, only HM achieved high predictions in C1 (0.66, 0.22). After eliminating IN impacted SF using Spearman’s rank-order correlation coefficient, a mean decrease in the C-I and MSE of 0.05 and 0.03 was observed in all four sequences. Conclusion: The IN method impacted the predictive power of survival models; thus, performance is sequence-dependent.

## 1. Introduction

Radiomics, the extraction of features from medical images using data characterization algorithms, is an upcoming field of research expected to yield non-invasive surrogates for important molecular characteristics [1]. The high-dimensional database obtained can be used to create predictive models to help medical professionals make decisions about patient care, such as prognosis, diagnosis, and treatment outcome prediction [1]. Magnetic Resonance Imaging (MRI) has superior soft-tissue contrast, which allows for increased sensitivity and specificity in disease detection [2]. Several MR-based radiomics studies have been performed to determine image biomarkers that can help assess prognosis and improve treatment decisions [3,4,5,6]. Robust radiomics models often require large amounts of data; therefore, MR images are usually collected from multiple centres, sites, and scanners [7]. MR intensities are acquired in scanner-dependent arbitrary units, which leads to scans coming from different scanners and subjects not directly comparable, even when the same scanning protocol is implemented [8,9]. While this intensity variation has no major effects on the clinical diagnosis, it drastically impacts the performance of subsequent MRI preprocessing, such as image registration and segmentation, as well as radiomics feature calculation [10,11,12]. Therefore, intensity normalization methods should be implemented to deal with the intra- and inter-scan image intensity variations. This task has nonetheless been proven non-trivial, as speed, accuracy, and applicability can vary depending on the given data set. Multiple intensity normalization algorithms have been developed over time [13,14,15,16,17,18]. However, even though the image biomarker standardization initiative (IBSI) has defined a more general standardized radiomics image processing workflow, no specific guidelines on the proper choice of intensity normalization methods are currently present [19].

In the study of survival prediction in high-grade glioma patients using MR-based radiomics and deep learning, 23 publications were analyzed. The methods identified are z-score transformation or similar (30%), histogram-matching [13] (27%), the MR brain-specific white strip [20] (18%), tissue-based [21] (3%), and feature-based [22] (1%) [23,24,25,26,27,28,29,30,31,32,33,34,35,36,37,38,39,40,41,42,43,44,45,46,47]. Intensity normalization was not reported in the remaining studies (Supplementary Table S1). Furthermore, a single intensity normalization method was applied to all MR sequences. With the rise of published radiomics studies, the issue of reproducibility has become more prominent [48,49]. Therefore, more effort is being put into investigating the effects of different imaging preprocessing steps on the overall robustness and reproducibility of the radiomics models [50,51,52]. Most of these investigations have targeted grey-level discretization, i.e., the clustering of pixels based on intensity values to reduce feature calculation time and noise. Nonetheless, Carre et al. showed that the reproducibility of radiomics features is influenced by grey-level discretization and the normalization method chosen [53]. Noting that radiomics features can be split into four different groups, i.e., shape-based, first-order, second-order [54], and higher-order statistics [55], the authors also report that z-score transformation of first and second-order radiomics features show robust results. In a systematic review of intensity normalization of MRI prior to radiomics feature extraction in glioma datasets, Fatania et al. report that intensity normalization is a key preprocessing step in developing robust radiomics signatures and that few comparative studies of multiple methods exist [56]. Um et al. studied the effect of intensity normalization in radiomics survival model prediction [57]. They found that using histogram matching to normalize MR intensity in primary glioblastoma (GBM) patients improved patient stratification by reducing radiomics feature variability in T1 weighted (w), T1w post-contrast agent (T1wce), and fluid-attenuated inversion recovery (FLAIR) scans. The radiomics signature consisted of a combination of all three radiomics feature groups. Li et al. performed an in vitro and in vivo study by applying seven different normalization methods on T1w images. They demonstrated that the feature-based harmonization method Combat [22] significantly removes scanner effects in brain MR-based radiomics studies [58].

Our study builds upon the studies mentioned above. It aims to investigate the use of different normalization algorithms in multi-scanner brain MRI datasets and, more precisely, the performance of different methods on different sequences and their impact on the survival prediction model’s performance by analyzing the predictive power of the methods’ respective normalized dataset in the prediction of overall survival (OS). Noting that the radiomics survival prediction signatures identified in previous work included features from all radiomics feature groups [23,25,26,27,29,30,31,32,33,34,35,36,37,38,39,40,41,42,43,44,45,46,47,59], our analyses were directly performed on the signature obtained through two multi scanners high-grade glioma (HGG) datasets after applying a stringent feature selection pipeline. The first cohort is a recurrent HGG (rHGG) cohort of 197 patients (C1), and the second is a primary HGG (pHGG) cohort of 141 patients (C2). The MR sequences considered are T1w, T1wce, T2w, and FLAIR. Significant OS-correlated features were first identified through multiple feature reduction and resampling techniques from MR images acquired pre-radiotherapy (RT) and normalized through 15 different normalization approaches. Sequence-specific survival radiomics prediction models were next trained using Cox proportional hazard and Poisson survival regressions and applied to both cohorts. The performances of the intensity normalization algorithms were then compared based on the predictive power of their respective normalized dataset in the predictions of OS. Finally, features affected by the intensity normalization methods were further rejected, and comparisons with models trained with the remaining significant features were performed.

## 2. Materials and Methods

### 2.1. Datasets

This study analyses multiparametric pre-RT MR sequences from two independent data cohorts. Patients’ eligibility was based on the availability of clinical information of at least two MR sequences taken no longer than 30 days before RT and of the RT-DICOM data, specifically the DICOM structure set (SS). The first cohort (C1) consisted of 197 patients with pathologically confirmed rHGG collected retrospectively from 15 different MR scanners at the Heidelberg Ion-Beam Therapy Centre (HIT) and University Clinic Heidelberg (UKHD) from 2009 to 2018. All 197 patients received particle irradiation. The second cohort (C2) consists of 141 pHGG patients collected retrospectively from 14 different MR scanners at the UKHD from 2011 to 2016. All 141 patients received standard photon RT. Patients between cohorts were matched for the frequency in gender, tumour grade, and MR sequence. OS was calculated as the number of days between the start of the re-RT (C1) or RT(C2) and death. MR scans were acquired post-surgical tumour resection prior to radiotherapy treatment (RT). Conventional multislice (2D) acquired in the axial, sagittal, or coronal plane and 3D scans are present. The MR sequences found in the cohorts are the widely used sequences for brain tumour imaging [60] in clinical routines and trials [61,62]. However, the classes considered in this study are T1w, T1wce, T2w, and FLAIR. The four considered sequences were identified at different rates in both cohorts. The in-plane resolution ranged from 0.45 × 0.45 to 1.40 × 1.40 mm in the discovery cohort and 0.33 × 0.33 to 2 × 2 mm in the test set. Slice thickness ranged from 0.9 to 5 mm in all MR scans. A summary of both cohorts is shown in Table 1. An overview of the MR scanners and protocols found are reported in Supplementary Tables S2 and S3.

### 2.2. MRI Preprocessing Workflow

**Figure 1:**
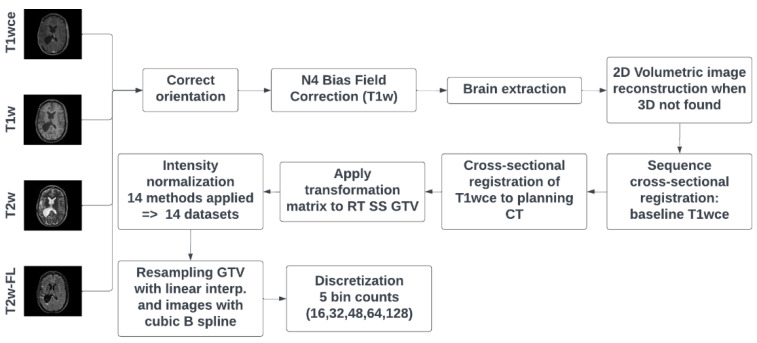
MR preprocessing diagram was applied to both cohorts. RT SS GTV represents the gross tumour volume segmentation extracted from the DICOM RT structure set. T2w-FL: T2w-FLAIR.

DICOM dataset curation and MR image classification were performed using pyCuRT and MR-Class [63]. All images were first reoriented to a common orientation. T1w images were corrected for signal inhomogeneities using the N4 bias field correction algorithm [64]. Brain extraction with the HD-BET tool was next performed [65]. When available, 3D MR sequences were mainly selected. Motion correction and volumetric image reconstruction were performed when 2D transversal, sagittal, and coronal MR scans were present. Reconstruction of the low-resolution 2D slices to a high-resolution 3D MR was performed using NiftyMic [66]. Next, cross-sectional linear co-registrations with six degrees of freedom (DOF) of the present MR images were performed on the T1wce using advanced normalization tools (ANTs) [67]. Furthermore, cross-sectional linear co-registrations with 6 DOF of the T1wce were performed on the RT planning CT. This registration was solely to generate the MR to CT transformation matrix, used to bring the target volume (TV) segmentations extracted from the DICOM SS objects to the MR space. Next, intensity normalization was performed. The different intensity normalization methods implemented in the comparison study are described in the next section. All images and segmentations were then resampled to a matrix size of 2 × 2 mm and a slice thickness of 2 mm using a cubic spline and linear interpolation, respectively. As for image discretization in an attempt to neutralize the impact of grey-level discretization on the overall result, five different bin counts were implemented, resulting in five sets of features per normalization algorithm. A bin count discretization approach was implemented since it was more frequently seen in HGG radiomics survival prediction studies. The image preprocessing diagram is shown in Figure 1.

### 2.3. Intensity Normalization Methods

Intensity normalization was performed with the help of the intensity normalization package by Reinhold et al. [68] and the FMRIB’s Automated Segmentation Tool (FAST) [69]. The intensity-normalization methods considered are: Fuzzy C-Means (FCM) [21] (9 different masks combinations), kernel density estimation (KDE), Gaussian mixture models (GMM) [70], Nyul’s and Udupa’s histogram matching-based abbreviated in this study as HM [13], white-strips (WS) [20], z-score normalization, and the feature-based batch adjustment method, i.e., Combat [22], resulting in 15 different MRI normalized datasets. A brief description of the methods is given in this section. For a broader description, we refer to the original normalization method papers and the manuscript by Reinhold et al. [68].

#### 2.3.1. Standard Score

The standard score, also known as the z-score, represents the distance of a raw score from the mean measured in standard deviations. In MR brain image normalization, given that B is the brain mask in image I, the z-score calculates the mean μ and standard deviation σ of the intensities inside the brain image (excluding the background) as follows: $\mu =\frac{1}{\left|B\right|}*\sum_{b\in B}^{\;}\;I\left(b\right),$ $\sigma =\sqrt{\frac{\sum_{b\in B}^{\;}\;{\left(I\left(b\right)-\mu \right)}^{2}}{\left|B\right|-1}}$ with the normalized image being $I_{\mathrm{norm}}\left(x\right)=\frac{I\left(x\right)-\mu }{\sigma }$. A disadvantage of this method is that the high intensities in the images are usually attenuated, risking a loss of information.

#### 2.3.2. Fuzzy Clustering

Clustering is a method for analyzing data that aims to discover structures or groups in a data set. Fuzzy clustering allows a piece of data to be part of more than one cluster. In a fuzzy c-means algorithm, a data point is assigned a membership function, with 0 being the farthest from a cluster’s centre and one being the closest to a cluster’s centre, with the data point theoretically being able to belong to all clusters. Used as a normalization technique in brain MRI, the fuzzy c-means algorithm uses the segmentation of specific brain tissue to normalize the image to the mean intensity of the tissue. If the tissue mean is $\mu =\frac{1}{\left|T\right|}*\sum_{t\in T}^{\;}\;I\left(t\right)$, then the normalized image is $I_{\mathrm{norm}}\left(x\right)=\frac{I\left(x\right)}{\mu }$, where x is the image voxels, and T is the tissue mask. The segmentations of the brain tissue masks, i.e., white matter (wm), grey matter (gm), and cerebrospinal fluid (csf), were performed using FSL’s FAST. In conjunction with the most common intensity value (mode) in a particular image, nine different mask combinations were implemented to generate nine fuzzy c-means normalized datasets. The masks are csf, gm, wm, csf-gm, wm-csf, wm-gm, csf-mode, wm-mode, and gm-mode. The normalization with two brain tissue masks is performed as: With µ_1_$=\frac{1}{\left|T1\right|}*\sum_{t\in T1}^{\;}\;I\left(t\right)$ and µ_2_ $=\frac{1}{\left|T2\right|}*\sum_{t\in T2}^{\;}\;I\left(t\right)$ the normalized image is derived as I_norm_(x) = $\frac{I\left(x\right)-a}{b-a}\;$ with a = min(µ_1_, µ_2_) and b = max(µ_1_, µ_2_) The normalization with a brain tissue mask and the mode is performed as: as I_norm_(x) = $\frac{I\left(x\right)}{\mathrm{diff}}\;$ with diff = µ_T_ − mode(B) with T as the tissue mask and B as the brain mask.

#### 2.3.3. Kernel Density Estimation

A density estimator aims to find a function for the probability distribution from which a dataset is generated. The kernel density estimation (KDE) is an empirical calculation in a parametrized form. The formula for calculating the KDE for the probability distribution function is $p\left(x\right)=\frac{1}{N*M*L*h}*\sum_{i=1}^{N*M*L}\;K\left(\frac{x-x_{i}}{h}\right)$, where N, M, and L are the sizes of the images, K is the kernel (normalized to one), and h is the bandwidth parameter that scales the kernel. This method provides a smoother version of the histogram, making it easier to find the maxima π, which is used to normalize the entire image as $I_{\mathrm{norm}}\left(x\right)=\;c\;*\;\frac{I\left(x\right)}{\pi }$, where c is a positive, real constant. For the MR brain images, the KED finds the peak of the white matter histogram and translates it to a standard value.

#### 2.3.4. Mixture Models

A mixture model assumes that a data set comprises subsets whose individual distributions are the respective probability distributions in the overall data set. A specific mixture model is the Gaussian mixture model, where the subsets are considered to be generated from a finite number of Gaussian distributions with undefined parameters. The method used in our study fits three Gaussian distributions to the histogram of the brain mask and normalizes the white matter mean to a standard value.

#### 2.3.5. Landmark-Based Histogram Matching

The landmark-based histogram matching method by Nyúl et al. deforms the input image intensity histogram to match a reference histogram. The reference histogram is commonly obtained by averaging histograms in a data set and setting the landmarks of interest. Each input image histogram is then matched to the reference through linear interpolation based on the defined landmarks, usually quantiles.

#### 2.3.6. White Stripe Normalization

The white stripe normalization approach by Shinohara et al. normalizes an image based on the normal-appearing white matter (NAWM) [20]. The NAWM values are obtained through a smoothening of the image histogram, followed by selecting the largest peak μ. The so-called white stripe contains intensity values up to 10% around μ. The white stripe can be defined as $\Omega_{T}=\left\{I\left(x\right)\;|{\;F}^{-1}\left(F\left(\mu \right)-\;\tau \right)<\;I\left(x\right)<{\;F}^{-1}\left(F\left(\mu \right)+\;\tau \right)\right\}$, where F(x) is the cumulative distribution function of the image I and τ = 5%. If σ is the standard deviation in the white stripe, the normalized image is I_norm_(x) = $\frac{I\left(x\right)-\;\mu }{\sigma }$.

#### 2.3.7. Combat

Combat is a feature-based method originally developed for microarray expression data [22]. However, it has also been applied in imaging data and radiomics studies in recent years [71,72,73]. It eliminates batch effects through a known batch covariate using parametric or non-parametric empirical Bayes frameworks. In this study, an empirical Bayes Combat method was applied through the Surrogate Variable Analysis (sva) package (v 3.20.0) to eliminate batch effects due to the MR scanner. Adjustment of the following covariates was performed: age, tumour grade, and gender.

### 2.4. Comparison Study Design

After MR image preprocessing, radiomics features were calculated automatically from the gross tumour volume (GTV) segmentations extracted from the DICOM RT structure set and the original image, as well as from derived images from each normalized/discretized dataset using Pyradiomics (v 3.0) [74]. The derived images were retrieved from first Wavelet filtering, which yielded eight decompositions per level, each representing a combination of either a high or a low pass filter in each of the three dimensions, and then by applying a Laplacian of Gaussian filter with spatial scaling factors (SSFs) of 2, 3, and 4 mm. The total yielded features were around 1200 per MR sequence. The different feature classes and corresponding feature numbers can be seen in Table 2.

**Figure 2:**
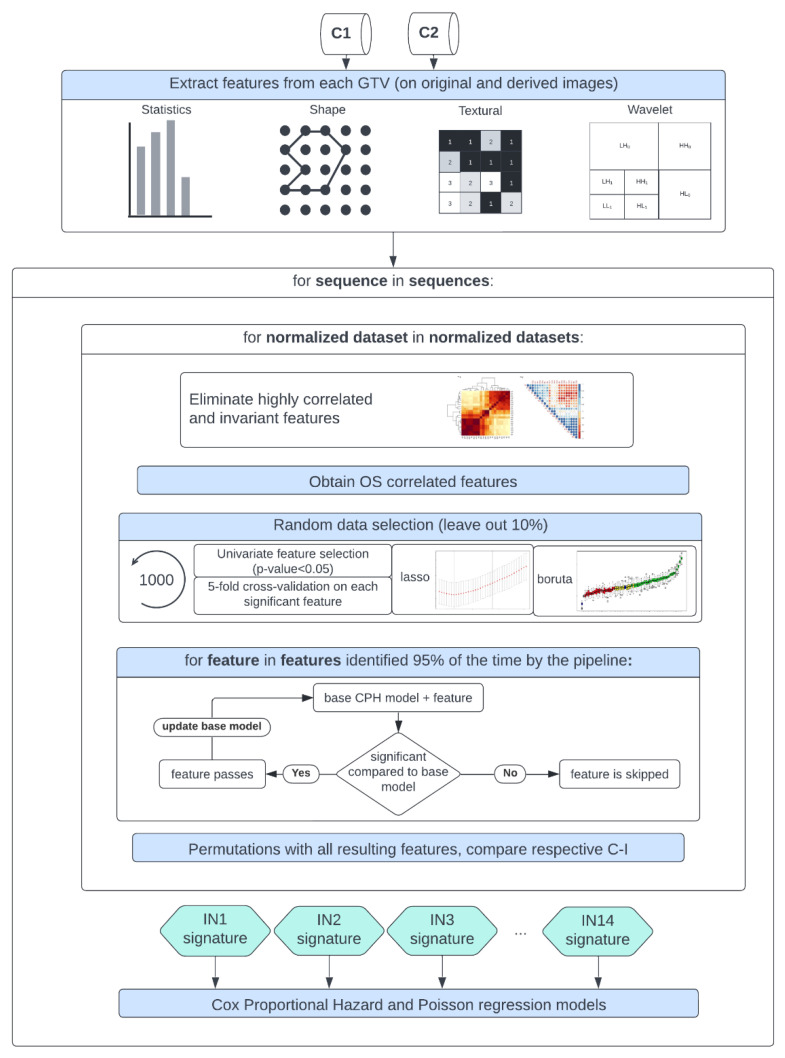
Study workflow—following MR image preprocessing on cohorts C1 and C2, features were extracted from each normalized dataset, intensity normalization method-specific radiomics signatures were derived, and Cox proportional hazards and Poisson regression models were trained.

A Spearman rank-order correlation coefficient was used on the total number of features to exclude redundant features (rs > 0.80). Three feature selection methods, including a univariate analysis under Cox proportional hazard (CPH) models (*p* < 0.05), a random forest (RF)-based method, and lasso regression, were applied on 1000 random subsamples of C1 and C2 (10% left out) separately to identify features correlated to OS. Sequence-specific significant features identified at least 950 times were selected, and survival analyses were conducted using CPH [75] and Poisson survival regressions (POI) models [76]. A ranking score was next assigned to each normalization approach based on the converted standardized z score of the CPH averaged 10-fold cross-validated (CV) concordance index (C-I), the POI averaged 10-fold CV mean square error (mse), and the respective Akaike information criterion (AIC) of the OS prediction models. Lastly, after identifying the top-ranked methods for the different MR sequences, correlation heatmaps between the different normalization approaches for each significant feature forming the sequence-specific radiomics signature were plotted. Stable features that showed a high correlation (rs > 0.80) between at least 12 intensity normalization methods were further used to train CPH and POI models again, and the effects on the model predictions were studied. Finally, the performance of the feature-based method Combat was assessed in combination with the top-ranked image-based normalization method for each sequence in both cohorts. A flowchart of the study design is shown in Figure 2.

## 3. Results

### 3.1. Performance Assessment of the Intensity Normalization Method-Specific Survival Prediction Models for the Different MR Sequence

**Figure 3:**
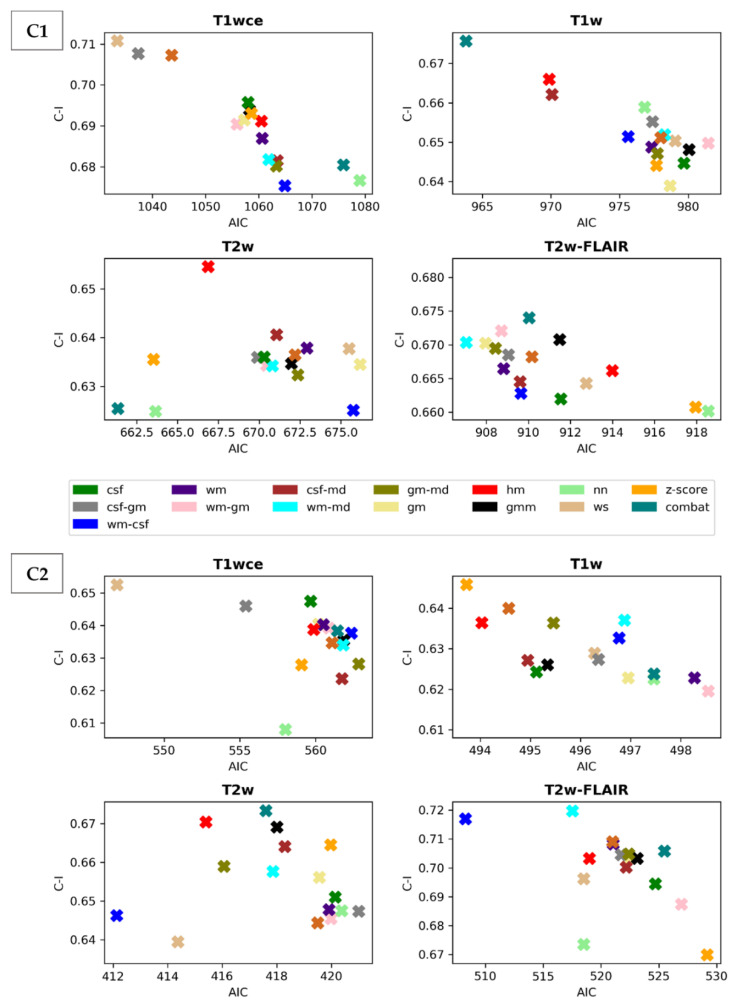
Scatter plots of the averaged (over the five bin counts considered) C-index vs AIC obtained by the CPH models for all four sequences in the study. Upper panel: cohort C1, Lower panel: cohort C2. csf: cerebrospinal fluid, wm: white matter, gm: grey matter, md: mode, gmm: Gaussian mixture models, kde: kernel density estimation, hm: Nyúl/Udupa histogram matching, ws: white stripe, nn: no normalization.

**Figure 4:**
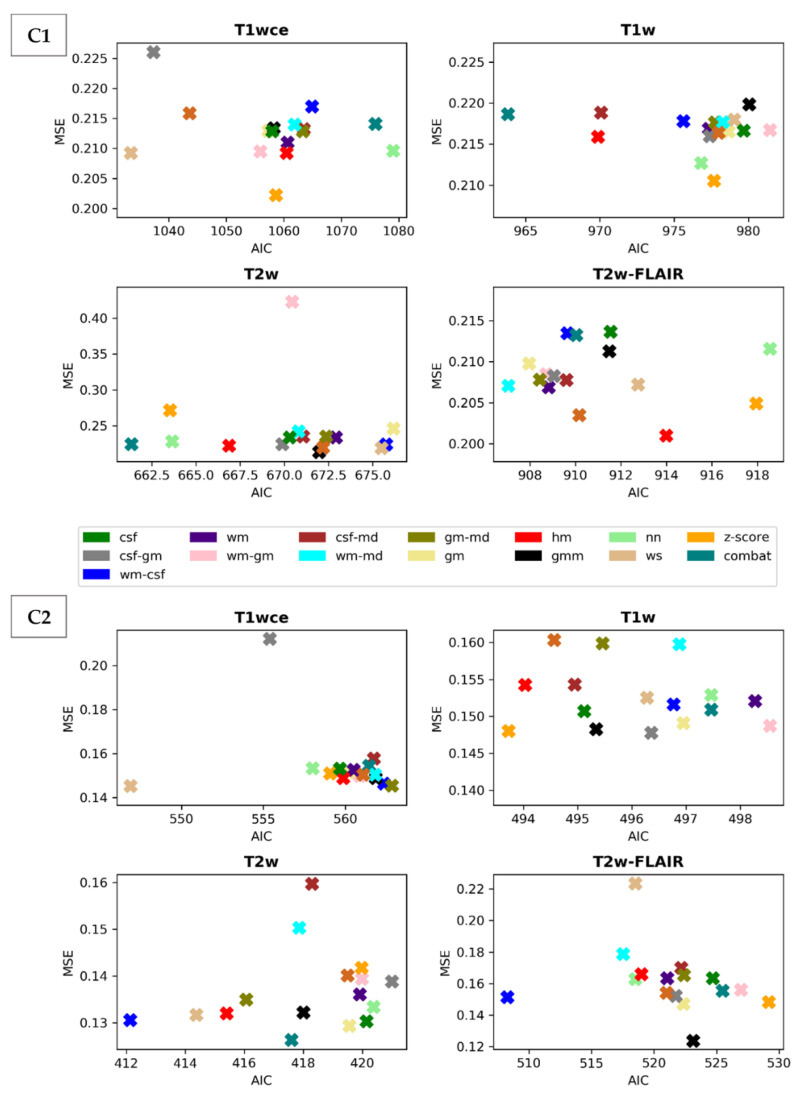
Scatter plots of the averaged (over the five bin counts considered) mse vs AIC obtained by the POI models for all four sequences in the study. Upper panel: cohort C1, Lowe panel: cohort C2. csf: cerebrospinal fluid, wm: white matter, gm: grey matter, md: mode, gmm: Gaussian mixture models, kde: kernel density estimation, hm: Nyúl/Udupa histogram matching, ws: white stripe, nn: no normalization. Table 2 summarizes and ranks the performance scores of the intensity normalization methods for each of the four MR sequences considered in both cohorts.

Scatter plots of the CPH averaged (over the five bin counts investigated) C-index and POI averaged mse, plotted against the respective AIC, for the 15 different intensity normalization-specific OS models derived from cohorts C1 and C2 are shown in Figure 3 and Figure 4. The OS model derived from the non-normalized (nn) dataset is also plotted.

Due to result variations and for a better interpretation of the performance of the different intensity methods, a ranking score was assigned to each normalization approach based on the converted standardized z score of the CPH averaged 10-fold CV C-I, the POI averaged 10-fold CV mean mse and the respective AIC of the OS prediction models. Table 3 summarizes and ranks the performance scores of the intensity normalization methods for each of the four MR sequences considered in cohorts C1 and C2.

The white stripe method is ranked first for T1wce in both cohorts (C1/C2 10-fold CV C-I: 0.71/0.65, AIC: 1033/547, 10-CV mse: 0.21/0.14, AIC: 410/252). For T1w, the feature-based batch adjustment method, i.e., Combat, had the best performance in C1 (0.68, 964, 0.22), while z-score transformation in C2 (0.65, 494, 0.15, 239). Nevertheless, the HM method was ranked second for both cohorts (C1/C2, 0.66/0.64, 970/494, 0.21/0.15, 389/2371). Furthermore, the top two ranked methods for T2w in both cohorts were Combat (C1/C2 0.62/0.67, 661/417, 0.22/0.13, 292/199) and the HM method (C1/C2 0.65/0.67, 667/415, 0.22/0.13, 294/200). As for T2w-FLAIR, the Fuzzy C-Means algorithm showed the best performance in C1 and C2, however, with different masks. For C1, the mask combination of wm and mode (0.67, 907, 0.21, 366) had the best performance, while the mask combination of wm and csf (0.72, 508, 0.15, 230) showed the best results for C2. Nevertheless, the former was ranked second in C2 (0.72, 517, 0.18, 235). Performance metrics of the remaining models in both cohorts are summarized in Supplementary Tables S4 and S5.

### 3.2. Significant Feature Correlation between the Normalized Datasets

**Figure 5:**
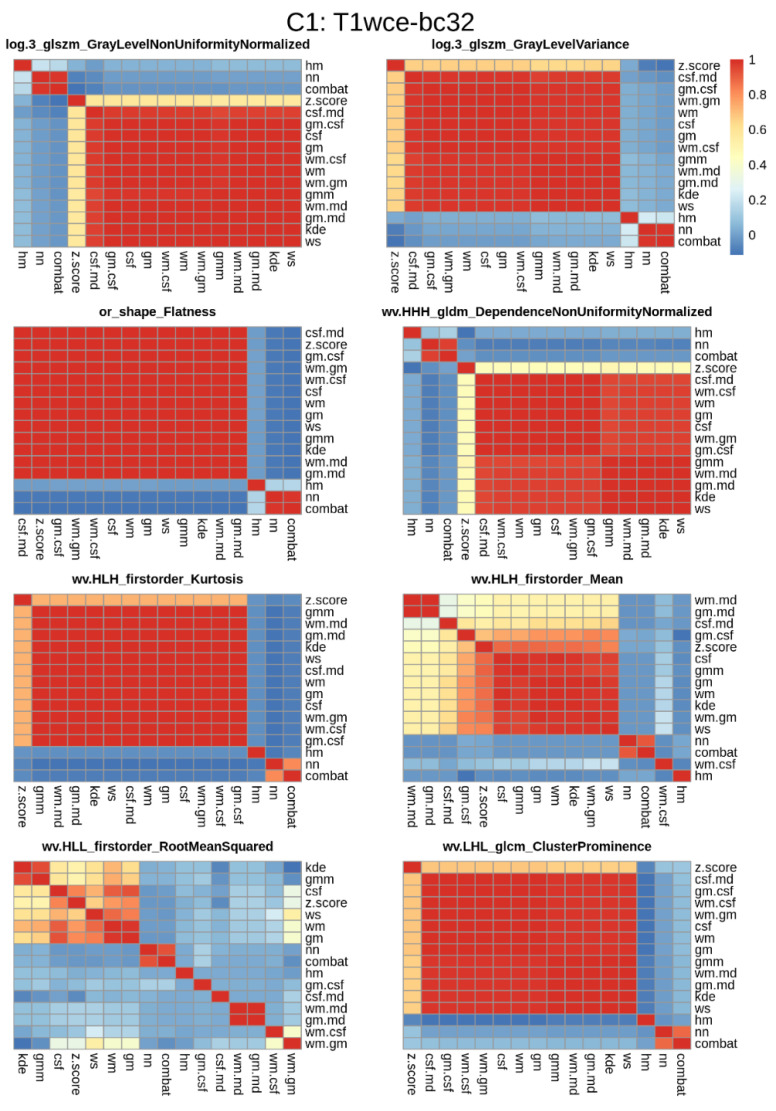
Significant feature correlation heatmaps between the 15 different normalization methods considered and the reference non-normalized dataset for T1wce images from cohort C1 discretized with a bin count of 32. Features with a high correlation (rs > 0.8) between at least 12 methods were further selected for modelling. Wv: Wavelet filter transformation, or: original image, log 3: Laplacian of gaussian transformation with a sigma of 3 mm.

Pairwise correlation tests were conducted to determine which features are impacted by the intensity normalization methods. Heatmaps displaying the Spearman correlation between the significant features and normalization methods for the various bin counts were generated. An example of T1wce significant features from C1 and bin count 32 is shown in Figure 5. The remaining heatmaps can be seen in Supplementary Figure S1.

**Figure 6:**
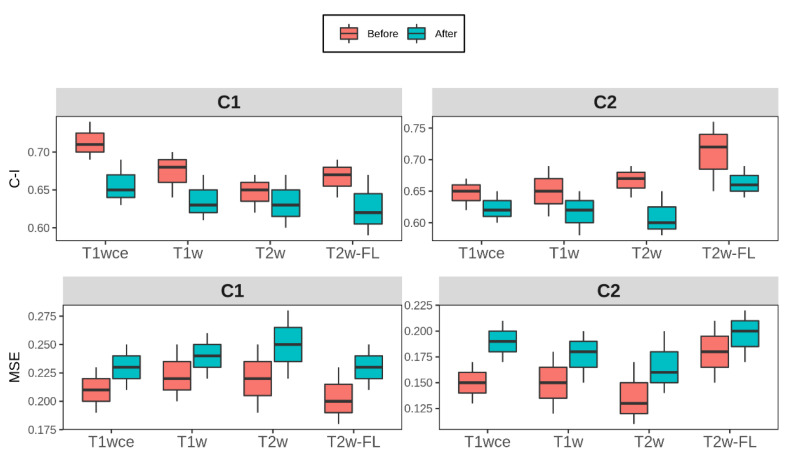
Box plots of the top-ranked image normalization method evaluation metrics C-I and MSE before and after the elimination of the intensity normalization impacted significant features for cohorts C1 and C2 for each MR sequence. The average (across all bin counts) 10-CV C-I/MSE with 95% confidence intervals are plotted. Performance of both models was similarly affected after the elimination of the intensity normalization impacted significant features, with a mean decrease in the 10-CV C-I and 10-CV MSE of 0.05 and 0.03 in all four sequences across both cohorts C-I: Concordance-index, mse: mean squared error, T2w-FL: T2w-FLAIR.

Although all features were found to be significantly correlated with OS, some feature distributions varied when different intensity normalization methods were applied. To assess the performance of the top-ranked image normalization method before and after the elimination of the intensity normalization impacted significant feature for cohort C1 and C2 for each MR sequence, the 10-CV C-I and mse of the CPH and POI models with only the stable features that have a high correlation (rs > 0.8) between at least 12 methods are reported (Table 4). Figure 6 displays boxplots showing the differences in C-I and MSE before and after the elimination of the intensity normalization impacted significant features for each modality in both cohorts.

### 3.3. Performance Comparison of the Feature-Based and Top-Ranked Image-Based Normalisation Methods

Table 5 summarizes the performance of the top-ranked image normalization method separate and in combination with the feature-based method Combat for cohorts C1 and C2. Since Combat ranked first for the T1w models from C1 and T2w models from C2, the second-ranked method, i.e., the HM method, was the image-based intensity normalization method for these two datasets.

## 4. Discussion

This study evaluated the impact of MRI intensity normalization algorithms on MR-based radiomics survival prediction models in primary and recurrent high-grade glioma. The sequences considered are T1w, T1wce, T2w, and T2w-FLAIR. Performance assessment of the intensity normalization method-specific CPH and POI survival prediction models showed an impact on the survival predictions between the different intensity normalization methods and the different MR sequences. Therefore, it can be concluded that the MR intensity normalization approach directly impacts the overall power of the radiomics-based MR predictive models. Moreover, considering the variability of the acquired results for the different MR sequences, it can be seen that the intensity normalization algorithm performance is correlated with the MR sequence and that the problem cannot be simplified to one intensity normalization method.

Due to these variations and for a better interpretation of the results, the ranking score was developed. The WS method showed promising results in T1wce models as it was ranked first in two independent multi-scanner datasets. Combat and the HM method showed consistent prediction results between the two cohorts for T2w models. These two methods were the top-performing methods for T1w in C1; however, only HM achieved high predictions in C2. Combat performance in C2 might be due to the higher number of batches and the number of images per batch, as 22% of T1w images in C2 were missing, making batch effect removal more challenging. As for T2w-FLAIR, the FCM showed favorable results in both cohorts; however, with different mask combinations, including the wm and csf or wm and mode. A tighter intensity range is observed in T2w-FLAIR than the other sequences, as csf signals are attenuated. These results might indicate that a mask-based normalization approach might be more favourable when dealing with images with tighter intensity ranges. The application of both an image-based and feature-based normalization method had little impact on the performance of the CPH and POI models. Exceptions were observed in the dataset where Combat was ranked first, i.e., T1w in C1 and T2w in C2.

As CPH models were part of the radiomics signature building pipeline, POI models were also trained to assess whether model performances were biased to CPH models. Comparably to CPH models, the impact of the intensity normalization methods was also observed in the POI models. Furthermore, the performance of both models was similarly affected after the elimination of the intensity normalization impacted significant features. A mean decrease in the 10-CV C-I and 10-CV MSE of 0.05 and 0.03 was observed in all four sequences across both cohorts. The use of a correlation coefficient between different normalization methods as a feature robustness check leads to a trade-off between model stability and the risk of eliminating important imaging biomarkers.

This study included two independent HGG cohorts collected from a single university hospital, UKHD. However, since the data cohorts included data between 2008 and 2019, 19 different scanners from three vendors with a 0.5 to 3.0 Tesla range were identified. Intensity normalization improved OS prediction in radiomics survival models as the non-normalized datasets generally ranked low in both cohorts. Therefore, the need for normalization is based on the number of scanners and image protocols, not just the number of centers. However, an exemption is seen in the T1w dataset in C1. This exception might be because many images in the T1w dataset from C1 were reconstructed using NiftyMic (as mostly 2D MR scans were present) and therefore preprocessed before applying the intensity normalization methods [66].

Since multiple MR scanners were found in both cohorts, where some have been withdrawn from clinical practice, phantoms could not be applied to assess the impact of the intensity normalization methods. Therefore, the hard endpoint OS was used in this study as a possible appropriate surrogate.

In literature, multiple intensity normalization methods have been reported in HGG radiomics studies, where all implemented the same method across all MR sequences [56]. However, as demonstrated in this study, the performance of the different methods varies. This study shows that the variations are big and that if reproducibility of the radiomics model is to be possible, the method of intensity normalization should be reported. Another way is to eliminate features impacted by the different normalization methods. When unstable features are impacted, the performance of the individual MR sequence prediction models is reduced, a necessary trade-off for stable radiomics models. However, combining multiple stable radiomics signatures from multiple MR sequences or modalities might mitigate that reduction and lead to high survival prediction models.

All in all, the main strengths of our study are as follows. First, we found that different intensity normalization methods produce varied results across different sequences. Therefore, to generate a good predictive model in the context of MRI radiomics, it may be necessary to apply different normalization methods to different sequences. Secondly, the methodology presented in this study, where features impacted by normalization methods are screened using Spearman pairwise correlations, can result in more generalizable models and facilitate the reproducibility of MRI-based radiomics models.

The following limitations exist in this work. The application of different preprocessing methods makes it generally hard to assess the impact of different normalization methods seamlessly. The changes in the radiomics values are as much affected by other preprocessing methods as image discretization or delineating the region of interest, which suggests that the application of intensity normalization alone may not be enough. In this study, we attempted to limit the effect of intensity discretization by applying five different bin counts and reporting the average score. Nevertheless, as demonstrated by many radiomics robustness studies, the overall performance and reproducibility of the radiomics models are indeed affected by choice of discretization approach [50,51,52]. Nevertheless, similarly to using correlation coefficient heatmaps between the different normalization methods to determine stable radiomics features, the same can be implemented across different bin counts or widths. Furthermore, all GTVs were segmented following institutional guidelines for RT treatment. Nonetheless, delineation variabilities are known to impact radiomics features, and the impact of intensity normalization and ROI segmentation should also be evaluated. As automatic tumour segmentation networks become more robust and popular, these inter-observer variabilities would be reduced, thus eliminating another layer of variability. However, as segmentation networks are also impacted by the intensity normalization method, future work will evaluate the performance of different normalization methods on automatic segmentation networks. Another limitation of our approach is the need for a sufficient amount of observation for the survival models since these models rely on having enough data to accurately reflect the underlying relationship between the predictors and the response variable. A lack of observations can lead to unreliable or unstable model estimates and increase the risk of overfitting.

Moreover, differences in the performance of the different IN methods across both cohorts can be possibly due to the alterations in the structure of intra-tumour heterogeneity, which differ between pHGG and rHGG, as well as the difference in the treatment of rHGG in comparison to pHGG, since the treatment of rHGG is not standardized as for pHGG, i.e., incorporating surgery, postoperative adjuvant RT, and adjuvant chemotherapy [77]. In addition, heterogeneity of cohorts, such as in MGMT methylation, IDH1/2 mutation, and 1p/19q deletion, can also lead to survival prediction differences [78]. More detailed studies are needed to assess the impact in more stringently defined cohorts.

## 5. Conclusions

Variations in the results for the different MR sequences showed that the intensity normalization method performance is sequence-dependent and directly impacts the predictive power of glioma survival models. Therefore, the documentation of the adapted normalization approach is highly recommended and necessary to enable the reproducibility of the MRI-based radiomics model. The methodology presented in this study can be further implemented in different entities to determine the stable radiomics features for signature building. Future work includes the study of additional sequences and anatomy sites. One major limitation of this study is the difficulty in solely evaluating the effect of a specific preprocessing method due to the application of various preprocessing techniques. Therefore, future work will involve assessing the various preprocessing methods using the methodology proposed in this study.

## Figures and Tables

**Table 1:** Descriptions of the data cohorts C1 and C2 considered in this study.

	C1		C2	
	n	%	n	%
Patients	197	100	141	100
Gender				
Male	120	61	86	61
Female	77	39	55	39
Age				
<50	84	64	47	33
50–69	105	53	73	52
≥70	8	17	21	15
Tumour grade				
III	71	36	34	24
IV	126	64	65	46
MR sequence				
T1wce	197	100	141	100
T1w	186	94	135	96
T2w-FLAIR	168	85	118	83
T2w	141	71	100	71

**Table 2:** The number of shape, first and second-order statistics derived per sequence and calculated on both the original and derived images.

Class	No. Features
First-order statistics	19
Shape-based (3D)	16
Second-order statistics	
Gray Level Co-occurrence Matrix	24
Gray Level Run Length Matrix	16
Gray Level Size Zone Matrix	16
Neighboring Gray Tone Difference Matrix	5
Gray Level Dependence Matrix	14

**Table 3:** Ranking with scores of the intensity normalizations of the MR sequences for cohorts C1 and C2. T2w-FL: T2w-FLAIR, IN: Intensity normalization method.

C1	T1wce		T1w		T2w		T2w-FL	
	IN	Score	IN	Score	IN	Score	IN	Score
1	ws	0.71	Combat	0.13	hm	0.27	wm-md	0.02
2	kde	−0.13	hm	−0.28	Combat	−0.03	wm-gm	−0.11
3	csf-gm	−0.20	csf-md	−0.90	z-score	−0.28	kde	−0.13
4	z-score	−0.48	nn	−1.00	gmm	−0.38	gm-md	−0.23
5	wm-gm	−0.85	z-score	−1.14	csf-gm	−0.61	gm	−0.24
6	csf	−0.97	csf-gm	−1.58	kde	−0.71	wm	−0.42
7	hm	−1.04	wm-csf	−1.65	nn	−0.76	csf-gm	−0.46
8	gmm	−1.11	wm	−1.85	csf	−0.78	Combat	−0.77
9	gm	−1.13	kde	−1.88	wm-md	−0.80	csf-md	−0.77
10	wm	−1.24	wm-md	−1.95	gm-md	−0.96	hm	−0.80
11	wm-md	−1.67	gm-md	−2.05	csf-md	−1.09	wm-csf	−1.01
12	csf-md	−1.71	ws	−2.15	ws	−1.18	gmm	−1.02
13	gm-md	−1.72	csf	−2.16	wm	−1.22	ws	−1.29
14	wm-csf	−2.16	gm	−2.23	wm-gm	−1.72	csf	−1.75
15	Combat	−2.25	wm-gm	−2.37	gm	−1.79	z-score	−2.21
16	nn	−2.27	gmm	−2.48	wm-csf	−2.01	nn	−2.65
C2								
1	ws	1.00	z-score	0.64	Combat	0.07	wm-csf	0.66
2	csf	−0.54	hm	−0.11	hm	−0.09	wm-md	−0.32
3	hm	−0.73	csf	−0.34	gm-md	−0.21	gmm	−0.56
4	z-score	−0.76	gmm	−0.35	wm-csf	−0.24	kde	−0.63
5	gm	−0.77	csf-md	−0.81	gmm	−0.41	csf-gm	−0.71
6	wm	−0.87	kde	−0.93	wm-md	−0.78	wm	−0.72
7	wm-gm	−0.87	gm-md	−0.97	gm	−1.00	gm	−0.76
8	csf-gm	−0.96	csf-gm	−0.97	csf-md	−1.12	hm	−0.81
9	kde	−0.98	ws	−1.04	ws	−1.13	gm-md	−0.90
10	wm-csf	−1.07	gm	−1.18	z-score	−1.21	csf-md	−1.05
11	gmm	−1.10	Combat	−1.20	csf	−1.31	nn	−1.25
12	wm-md	−1.13	nn	−1.41	kde	−1.36	csf	−1.35
13	Combat	−1.19	wm-csf	−1.43	wm	−1.52	Combat	−1.42
14	gm-md	−1.28	wm-md	−1.64	nn	−1.60	ws	−1.50
15	csf-md	−1.39	wm-gm	−2.01	wm-gm	−1.69	wm-gm	−1.59
16	nn	−1.82	wm	−2.11	csf-gm	−1.81	z-score	−2.11

**Table 4:** Performance of the top-ranked image normalization method separate before and after the elimination of the intensity normalization impacted significant features for cohort C1 and C2 for each MR sequence. The average (across all bin counts) 10-CV C-I/MSE with 95% confidence intervals are reported.

	C1		C2	
	Before	After	Before	After
T1wce	0.71 [0.69 0.74]/ 0.21 [0.19 0.23]	0.65 [0.63 0.69]/ 0.23 [0.21 0.25]	0.65 [0.62 0.67]/ 0.15 [0.13 0.17]	0.62 [0.60 0.65]/ 0.19 [0.17 0.21]
T1w	0.68 [0.64 0.70]/ 0.22 [0.20 0.25]	0.63 [0.61 0.67]/ 0.24 [0.22 0.26]	0.65 [0.61 0.69]/ 0.15 [0.12 0.18]	0.62 [0.58 0.65]/ 0.18 [0.15 0.20]
T2w	0.65 [0.62 0.67]/ 0.22 [0.19 0.25]	0.63 [0.60 0.67]/ 0.25 [0.22 0.28]	0.67 [0.64 0.69]/ 0.13 [0.11 0.17]	0.60 [0.58 0.65]/ 0.16 [0.14 0.20]
T2w-FL	0.67 [0.64 0.69]/ 0.20 [0.18 0.23]	0.62 [0.59 0.67]/ 0.23 [0.21 0.25]	0.72 [0.65 0.76]/ 0.18 [0.15 0.21]	0.66 [0.64 0.69]/ 0.20 [0.17 0.22]

**Table 5:** Performance of the top-ranked image normalization method separate and in combination with the feature-based method Combat for cohort C1 and C2 for each MR sequence. The average (across all bin counts) 10-CV C-I/MSE with 95% confidence intervals are reported.

	C1			C2		
	Combat	I. Norm.	Combined	Combat	I. Norm.	Combined
T1wce	0.68 [0.66 0.70]/ 0.21 [0.19 0.23]	0.71 [0.690.74]/ 0.21 [0.19 0.23]	0.68 [0.66 0.69]/ 0.21 [0.19 0.23]	0.64 [0.62 0.68]/ 0.15 [0.13 0.17]	0.65 [0.62 0.67]/ 0.15 [0.13 0.17]	0.63 [0.61 0.66]/ 0.17 [0.15 0.19]
T1w	0.68 [0.64 0.70]/ 0.22 [0.20 0.24]	0.66 [0.64 0.68]/ 0.22 [0.19 0.24]	0.62 [0.59 0.64]/ 0.23 [0.20 0.26]	0.62 [0.60 0.66]/ 0.15 [0.12 0.17]	0.65 [0.61 0.69]/ 0.15 [0.12 0.18]	0.62 [0.59 0.65]/ 0.15 [0.11 0.16]
T2w	0.62 [0.59 0.64]/ 0.23 [0.21 0.23]	0.65 [0.62 0.67]/ 0.22 [0.19 0.25]	0.61 [0.58 0.63]/ 0.25 [0.23 0.27]	0.67 [0.64 0.69]/ 0.13 [0.11 0.17]	0.67 [0.64 0.69]/ 0.13 [0.11 0.15]	0.62 [0.59 0.65]/ 0.15 [0.13 0.19]
T2w-FL	0.67 [0.64 0.69]/ 0.21 [0.19 0.24]	0.67 [0.64 0.69]/ 0.20 [0.18 0.23]	0.64 [0.61 0.66]/ 0.24 [0.22 0.26]	0.70 [0.67 0.72]/ 0.16 [ 0.14 0.19]	0.72 [0.65 0.76]/ 0.14 [0.12 0.17]	0.68 [0.65 0.70]/ 0.17 [0.15 0.21]

## Data Availability

C1 and C2 are available from the corresponding author on reasonable request.

## Supplementary Materials

The following supporting information can be downloaded at: https://www.mdpi.com/article/10.3390/cancers15030965/s1, Table S1: Intensity normalization algorithms applied in MR-based radiomics and deep learning-based survival prediction studies in high-grade glioma patients. Table S2: MR scanner models found in the cohorts; Table S3: MR image protocols found in the cohorts; Table S4: Model performance metrics for each MR sequence and normalization method for cohort 1; Table S5: Model performance metrics for each MR sequence and normalization method for cohort 2; Figure S1: Correlation heatmaps between the 16 different normalization methods considered and the reference non-normalized dataset for each MR sequence in cohorts C1 and C2 discretized with bin counts of 16, 32, 48, 64, and 128.
