# Supplementary material for: MR Intensity Normalization Methods Impact Sequence Specific Radiomics Prognostic Model Performance in Primary and Recurrent High-Grade Glioma
Source: Cancers (Basel). 2023 Feb 2;15(3):965. doi: 10.3390/cancers15030965 (PMC9913466; doi:10.3390/cancers15030965)
Supplement: Supplementary file 1 [file cancers-15-00965-s001.zip › cancers-2179163-supplementary.pdf]

Abbreviations

AIC: Akaike Information Criterion
C-I: Concordance index
CPH: Cox Proportional Hazard models
CSF: cerebrospinal fluid
CV: cross-validation
DE: dependence entropy
DICOM: Digital Imaging COmmunication in Medicine
FLAIR: fluid attenuated inversion recovery
GBM: glioblastoma
GLCM: Gray Level Co-occurence Matrix
GLDM: Gray Level Dependence Matrix
GLRLM: Gray Level Run Length Matrix
GLSZM: Gray Level Size Zone Matrix
GM: gray matter
GMM: Gaussian mixture models
GTV: Gross Tumor Volume
HM: Nyul-Udupa histrogram matching
HR: Hazard ratio
KDE: kernel density estimation
LGLRE: Long Run Low Gray Level Emphasis
LRHGLE: Long Run High Gray Level Emphasis
LALGLE: Large Area Low Gray Level Emphasis
LoG: Laplacian of Gaussian
LoG3: Laplacian of Gaussian with sigma 3mm
MD: mode
MRI: magnetic resonance imaging
MSE: mean squared error
NN: no normalization
OS: overall survival
Or: original image
POI: Poisson regression models
RMS: Root Mean Squared

ROI: region of interest
rs: Spearman correlation coefficient
RSF: Random survival forest
RT: radiotherapy
SAE: Small Area Emphasis
SALGLE: Small Area Low Gray Level Emphasis
SE: sensitivity
SP: specificity
SRHGLE: Short Run High Gray Level Emphasis
SRLGLE: Short Run Low Gray Level Emphasis
SS: structure set
T1w: T1-weighted
T1wce: T1-weighted contrast-enhanced
T2w: T2-weighted
TI: Inversion time
TE: Echo time
TR: Repetition time
WM: white matter
WS: white stripe
WV: wavelet filter transformation

Tables and figures

Supplementary-Table S1. Intensity normalization algorithms applied in MR-based radiomics and deep learning-based survival prediction studies in high-grade glioma patients.

Method	Studies
Histogram matching	[24, 25, 31]
z-score	[26, 32, 33, 35, 37, 40, 43, 46]
White strip	[30, 45, 47]
No method/not mentioned	[23, 27, 29, 34, 36, 38, 41, 42, 44]

Supplementary-Table S2. MR scanner models found in the cohorts.

Dataset	Manufacturer	Tesla	Model
**C1**	Siemens	1	Harmony
		1.5	Avanto, Aera, Espree, Sonata, Symphony
		3	Prisma fit, Skyra, TrioTim,Verio
	Philips	1.5	Achieva, Ingenia, Intera
	GE	1.5	Optima MR450w, Signa HDxt
**C2**	Siemens	1	Allegra, Harmony
		1.5	Aera, Amira, Avanto, Espree, Vision
		3	Prisma fit, Skyra, Trio, TrioTim,Verio
	Philips	1	Panorama
		1.5	Achieva, Ingenia, Intera
	GE	1.5	Signa

Supplementary-Table S3. MR image protocols found in the cohorts. TI: Inversion time, TE: Echo time, TR: Repetition time, FA: Flip angle.

	Protocol	%	
**T1w**	Magnetization Prepared - RApid Gradient Echo (MPRAGE)	73	TI: 800-1100 ms, TE: 2.27-4 ms, TR: 1680-2200 ms, and FA: 7-15°<br>In-plane resolution: 0.42 x 0.42 – 1 x 1 mm, Slice thickness: 0.9-1.3 mm
	Spin Echo	12	TE: 8-17 ms, TR: 350-744 ms, and FA: 70-90°<br>In-plane resolution: 0.45 x 0.45 – 1.05 x 1.05 mm, Slice thickness: 3-6 mm
	Turbo Field Echo	4	TE: 3.17-4.7 ms, TR: 6.5-8.2 ms, and FA: 8°<br>In-plane resolution: 0.5 x 0.5 – 0.93 x 0.93 mm, Slice thickness: 0.9-2 mm
	3D FLASH	4	TE: 3.56-.9 ms, TR: 7.3-15 ms, and FA: 10-30°<br>In-plane resolution: 0.93x 0.93 mm, Slice thickness: 1.2 mm
	Turbo Spin Echo	2	TE: 11 ms, TR: 400-439 ms, and FA: 150°<br>In-plane resolution: 0.45 x 0.45 – 0.75 x .78 mm, Slice thickness: 3-6 mm
	FLASH	2	TE: 2.48-4 ms, TR: 220-355 ms, and FA: 70-90°<br>In-plane resolution: 0.4 x 0.4 – 0.6 x 0.6 mm, Slice thickness: 4-5 mm

	Fast Field Echo	1	TE: 1.69-2.41 ms, TR: 143-187 ms, and FA: 80°<br>In-plane resolution: 0.36 x 0.36 – 0.9 x 0.9 mm, Slice thickness: 5-6 mm
	Spoiled gradient echo (SPGR)	1	TE: 3.47 ms, TR: 8.8 ms, and FA: 12°<br>In-plane resolution: 0.47 x 0.47 mm, Slice thickness: 1.2 mm
	SPGR BRAVO	1	TI: 300 ms, TE: 5.2 ms, TR: 12.38 ms, and FA: 20°<br>In-plane resolution: 0.94 x 0.94 mm, Slice thickness: 1.2 mm
**T2w**	Turbo Spin Echo	86	TE: 10 ms, TR: 3000 ms, and FA: 140°<br>In-plane resolution: 0.22 x 0.22 – 0.97 x 0.97 mm, Slice thickness: 3-6 mm
	Multiple Spin Echo	7	TE: 10 ms, TR: 3000 ms, and FA: 140°<br>In-plane resolution: 0.88 x 0.88 mm, Slice thickness: 5 mm
	Fast Spin Echo	3	TE: 10 ms, TR: 3000 ms, and FA: 140°<br>In-plane resolution: 0.88 x 0.88 mm, Slice thickness: 5 mm
	Turbo Spin Echo/Propeller	2	TE: 100 ms, TR: 5150-6100 ms, and FA: 160°<br>In-plane resolution: 0.46 x 0.46 mm, Slice thickness: 5.mm
	Turbo Spin Echo/Blade	2	TE: 100 ms, TR: 4000 ms, and FA: 150°<br>In-plane resolution: 0.71 x 0.71 mm, Slice thickness: 5.5 mm
**T2w-FLAIR**	Fast FLAIR	69	TI: 1700 ms, TE: 95 ms, TR: 8000 ms, and FA: 90°<br>In-plane resolution: 0.35 x 0.35 mm, Slice thickness: 3 mm
	Turbo Dark Fluid	17	TI: 1950 ms, TE: 110 ms, TR: 9000 ms, and FA: 1500°<br>In-plane resolution: 0.94 x 0.94 mm, Slice thickness: 5 mm
	FLAIR with Fat Saturation (FS)	14	TI: 2500 ms, TE: 135 ms, TR: 10000 ms, and FA: 1800°<br>In-plane resolution: 1.05 x 1.05 mm, Slice thickness: 6 mm

Supplementary-Table S4. Model performance metrics for each MR sequence and normalization method for cohort C1 - AIC CPH: Akaike Information Criterion Cox Proportional Hazard models, AIC POI: AIC Poisson regression models, C-I: Concordance Index, mse: mean squared error, csf: cerebrospinal fluid, wm: white matter, gm: grey matter, md: mode, gmm: Gaussian mixture models, kde: kernel density estimation, hm: Nyúl/Udupa histogram matching, ws: white stripe, nn: no normalization.

C1	T1wce					T1w					T2w					T2w-FL				
	No. feat.	AIC CPH	AIC POI	C-I	mse	No. feat.	AIC CPH	AIC POI	C-I	mse	No. feat.	AIC CPH	AIC POI	C-I	mse	No. feat.	AIC CPH	AIC POI	C-I	mse
combat	4.4	1075.92	424.71	0.68	0.214	4	963.81	387.77	0.676	0.219	1.6	661.34	291.7	0.625	0.225	6	910.03	368.51	0.674	0.213
csf	6.6	1057.96	418.54	0.696	0.213	3.8	979.68	397.75	0.645	0.217	2.6	670.3	294.2	0.636	0.234	4.8	911.54	368.16	0.662	0.214
csf-gm	7.4	1037.33	410.22	0.708	0.226	4.2	977.4	396.51	0.655	0.216	3	669.88	295.01	0.636	0.225	5	909.05	366.76	0.669	0.208
csf-md	5.4	1063.44	421.71	0.682	0.213	3.6	970.08	391.51	0.662	0.219	2.4	671.05	295.44	0.641	0.235	5	909.6	366.94	0.665	0.208
gm	6.6	1057.27	419.28	0.691	0.213	4	978.68	397.49	0.639	0.217	2.8	676.17	297.95	0.635	0.246	5.4	907.96	365.91	0.67	0.21
gm-md	5.2	1063.3	421.46	0.68	0.213	4.2	977.74	397.17	0.647	0.218	2.2	672.36	295.78	0.632	0.235	5.4	908.43	366.3	0.669	0.208
gmm	6.4	1058.22	419.21	0.694	0.213	3.8	980.06	398.36	0.648	0.22	2.4	671.96	291.7	0.635	0.214	5.4	911.49	368.84	0.671	0.211
hm	6.6	1060.47	419.17	0.691	0.209	4.4	969.87	389.05	0.666	0.216	2	666.87	293.7	0.655	0.222	5.2	913.99	369.14	0.666	0.201
kde	6.4	1043.62	413.4	0.707	0.216	4.2	977.98	397.6	0.651	0.216	3	672.18	294.37	0.636	0.22	5.4	910.16	366.24	0.668	0.203
nn	4.4	1078.98	425.38	0.677	0.21	4.6	976.81	395.25	0.659	0.213	2.6	663.64	294.21	0.625	0.228	5.2	918.55	370.59	0.66	0.212
wm	6	1060.63	419.24	0.687	0.211	4.4	977.34	396.98	0.649	0.217	2.4	672.91	295.94	0.638	0.234	5	908.82	366.33	0.666	0.207
wm-csf	6	1064.88	423.03	0.675	0.217	4.4	975.62	394.81	0.651	0.218	3	675.76	298.16	0.625	0.224	4.6	909.64	364.39	0.663	0.213
wm-gm	6.4	1055.89	417.18	0.69	0.21	3.6	981.46	400.79	0.65	0.217	2.8	670.43	294.45	0.634	0.423	5	908.71	366.22	0.672	0.208
wm-md	5.4	1061.88	420.72	0.682	0.214	4.4	978.27	397.1	0.652	0.218	2.8	670.8	294.97	0.634	0.242	5.2	907.05	366.15	0.67	0.207
ws	6.8	1033.41	409.77	0.711	0.209	4.2	979.04	398.12	0.65	0.218	3	675.5	295.8	0.638	0.219	4.6	912.75	368.97	0.664	0.207
z-score	5.8	1058.63	416.4	0.693	0.202	3	977.69	394.24	0.644	0.211	2.4	663.53	293.13	0.636	0.272	4	917.95	371.92	0.661	0.205

Supplementary-Table S5. Model performance metrics for each MR sequence and normalization method for cohort C2 - AIC CPH: Akaike Information Criterion Cox Proportional Hazard models, AIC POI: AIC Poisson regression models, C-I: Concordance Index, mse: mean squared error, csf: cerebrospinal fluid, wm: white matter, gm: grey matter, md: mode, gmm: Gaussian mixture models, kde: kernel density estimation, hm: Nyúl/Udupa histogram matching, ws: white stripe, nn: no normalization.

C2	T1wce					T1w					T2w					T2w-FLAIR				
	No. feat.	AIC CPH	AIC POI	C-I	mse	No. feat.	AIC CPH	AIC POI	C-I	mse	No. feat.	AIC CPH	AIC POI	C-I	mse	No. feat.	AIC CPH	AIC POI	C-I	mse
combat	3.6	561.43	262.01	0.638	0.154	4.4	497.46	238.44	0.624	0.151	3.4	417.59	199.56	0.673	0.126	6	525.47	243.63	0.706	0.156
csf	3.2	559.66	258.3	0.647	0.153	3.2	495.12	236.39	0.624	0.151	3.8	420.13	199.39	0.651	0.13	6.4	524.68	239.19	0.694	0.164

csf-gm	2.8	555.38	255.67	0.646	0.212	4.4	494.94	238.34	0.627	0.154	3.6	421	201.05	0.647	0.139	6.8	521.74	236.78	0.705	0.152
csf-md	2.2	561.73	259.62	0.624	0.158	3.6	496.35	240.74	0.627	0.148	5	418.29	198.62	0.664	0.16	5.2	522.18	237.62	0.7	0.17
gm	5	560.18	259.12	0.64	0.151	3.2	496.95	239.6	0.623	0.149	4.8	419.55	200.26	0.656	0.129	6.6	522.35	237.27	0.703	0.147
gm-md	3.4	562.82	260.87	0.628	0.146	4	495.46	238.15	0.636	0.16	4.8	416.06	198.14	0.659	0.135	6.6	522.38	237.17	0.705	0.165
gmm	4	561.83	260.84	0.635	0.149	3.2	495.34	237.76	0.626	0.148	3.8	418	198.07	0.669	0.132	6.4	523.14	237.25	0.703	0.124
hm	2.6	559.87	258.8	0.639	0.149	2.8	494.03	237.45	0.636	0.154	3.4	415.39	200.42	0.67	0.132	5.8	518.98	237.71	0.703	0.166
kde	3.4	561.07	259.57	0.635	0.15	4.6	494.56	239.62	0.64	0.16	4.6	419.49	199.16	0.644	0.14	5.6	520.99	237.08	0.709	0.154
nn	3	558	264.03	0.608	0.153	3.2	497.46	238.44	0.623	0.153	3.8	420.37	199.49	0.648	0.133	8.4	518.5	236.25	0.674	0.163
wm	2.8	560.5	259.71	0.64	0.153	3	498.27	242.12	0.623	0.152	3.6	419.91	198.23	0.648	0.136	7.6	521.05	236.72	0.708	0.164
wm-csf	3.2	562.35	261.09	0.638	0.146	3.8	496.77	242.35	0.633	0.152	3.4	412.13	195.97	0.646	0.131	6	508.27	230.37	0.717	0.151
wm-gm	2.4	560.87	259.73	0.639	0.15	2.8	498.54	242	0.62	0.149	4	419.99	200.47	0.645	0.139	6.4	526.95	239.97	0.687	0.156
wm-md	2.8	561.8	260.5	0.634	0.151	3.8	496.87	240.59	0.637	0.16	4.6	417.86	198.19	0.658	0.15	5.4	517.53	234.81	0.72	0.179
ws	2.4	546.89	252.45	0.652	0.145	4	496.28	239.31	0.629	0.153	3.6	414.37	198.81	0.639	0.132	5	518.51	237.6	0.696	0.223
z-score	3.6	559.05	256.55	0.628	0.151	4.4	493.72	238.37	0.646	0.148	5	419.98	203.52	0.665	0.142	5.8	529.17	241.86	0.67	0.148

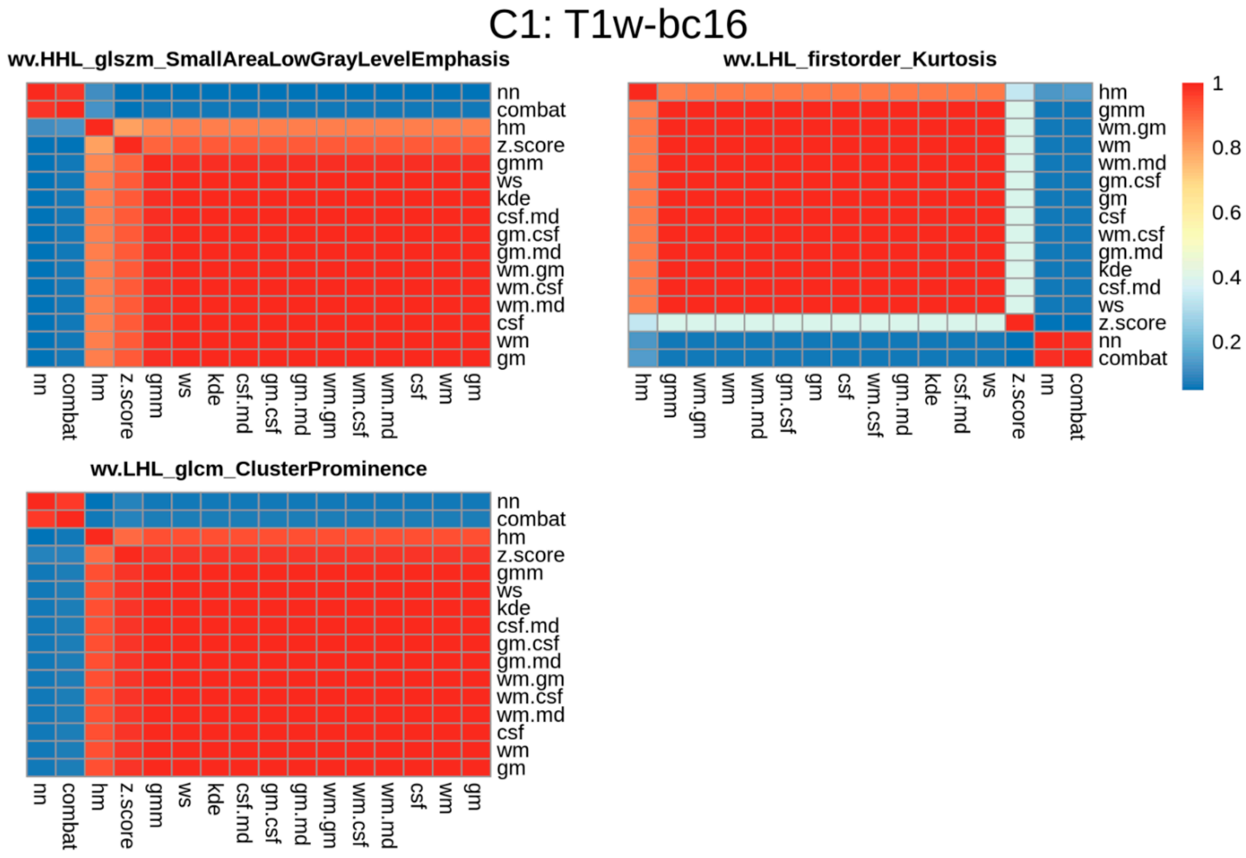

Supplementary Figure S1-1. Correlation heatmaps between the 15 different normalization methods considered and the reference non-normalized dataset for T1w significant features in cohort C1 discretized with bin counts of 16.

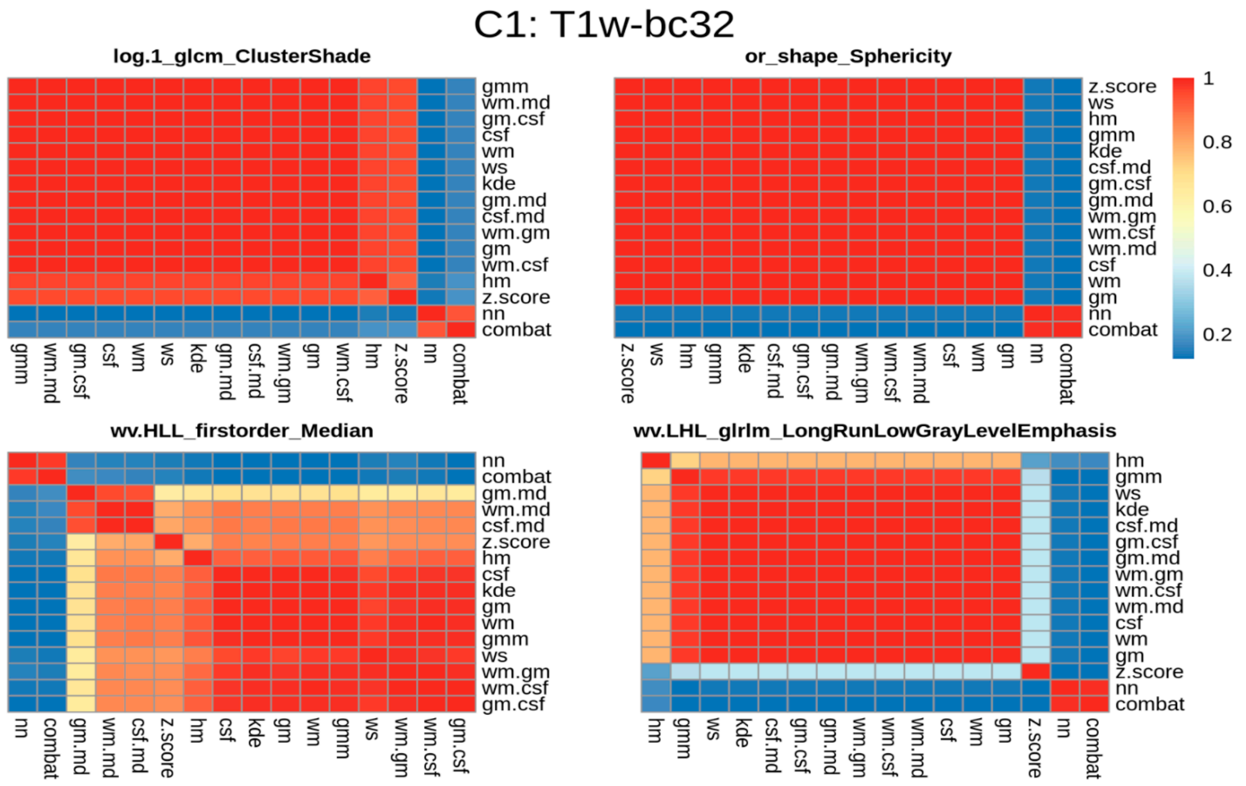

Supplementary Figure S1-2. Correlation heatmaps between the 15 different normalization methods considered and the reference non-normalized dataset for T1w significant features in cohort C1 discretized with bin counts of 32.

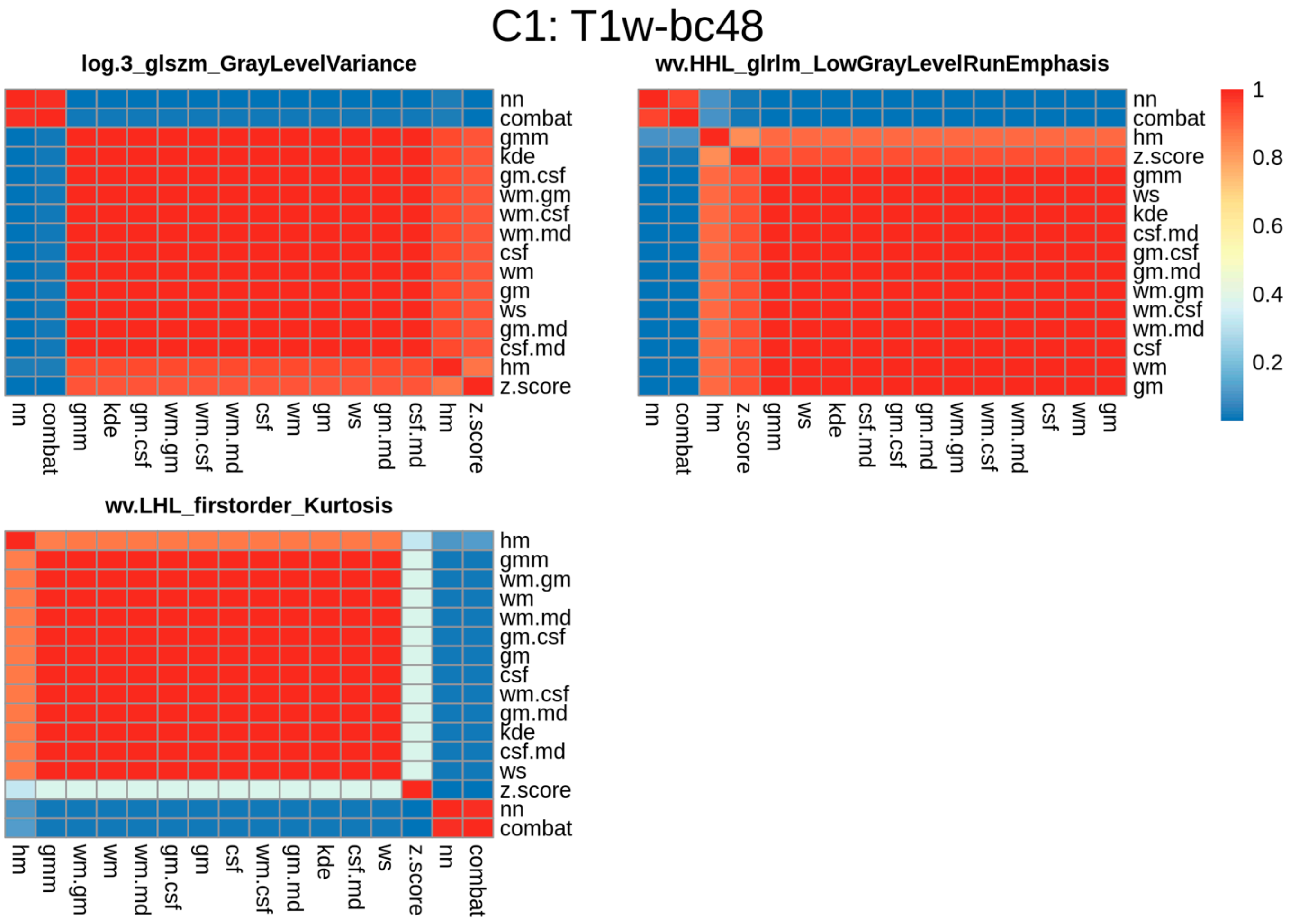

Supplementary Figure S1-3. Correlation heatmaps between the 15 different normalization methods considered and the reference non-normalized dataset for T1w significant features in cohort C1 discretized with bin counts of 48.

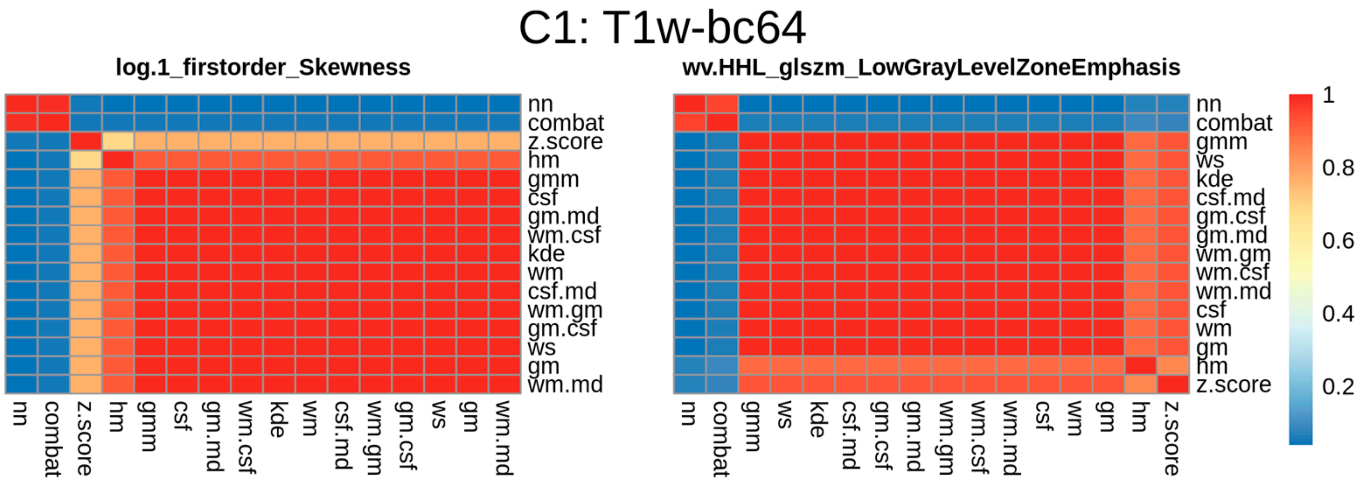

Supplementary Figure S1-4. Correlation heatmaps between the 15 different normalization methods considered and the reference non-normalized dataset for T1w significant features in cohort C1 discretized with bin counts of 64.

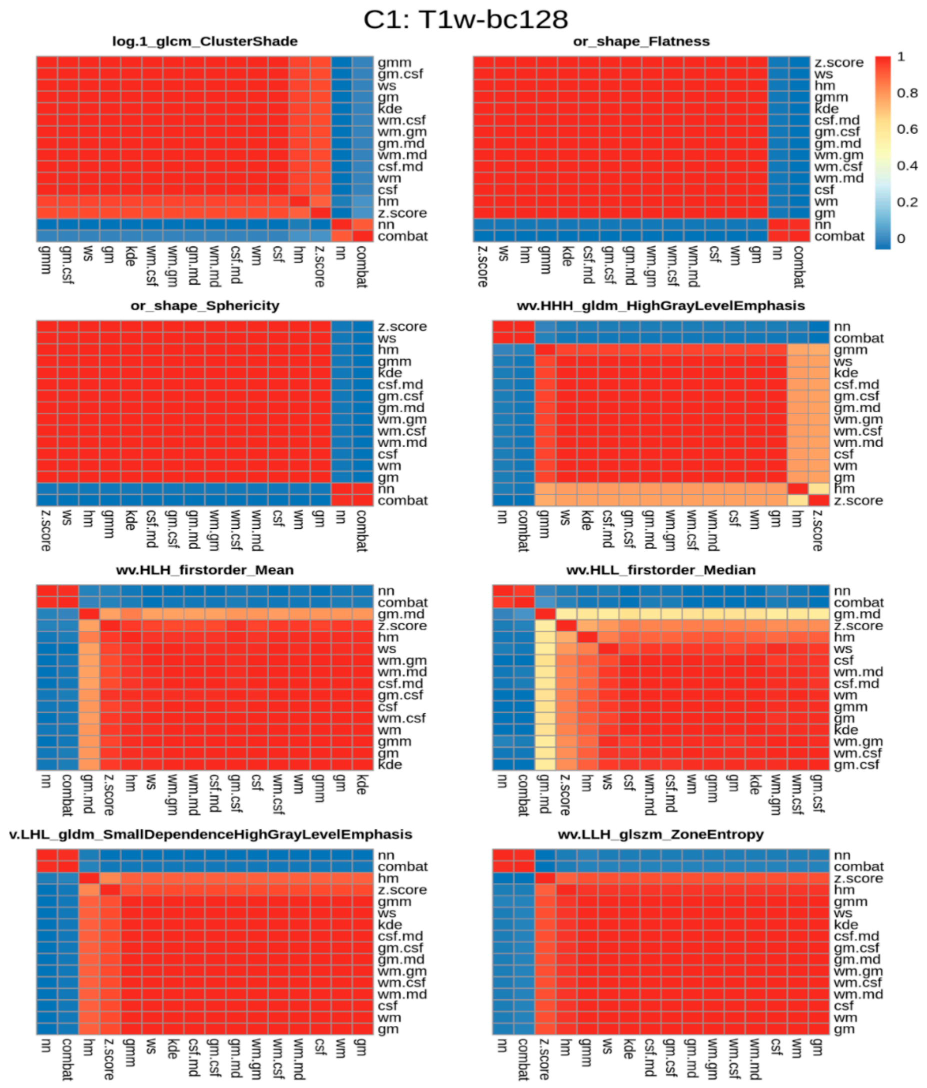

Supplementary Figure S1-5. Correlation heatmaps between the 15 different normalization methods considered and the reference non-normalized dataset for T1w significant features in cohort C1 discretized with bin counts of 128.

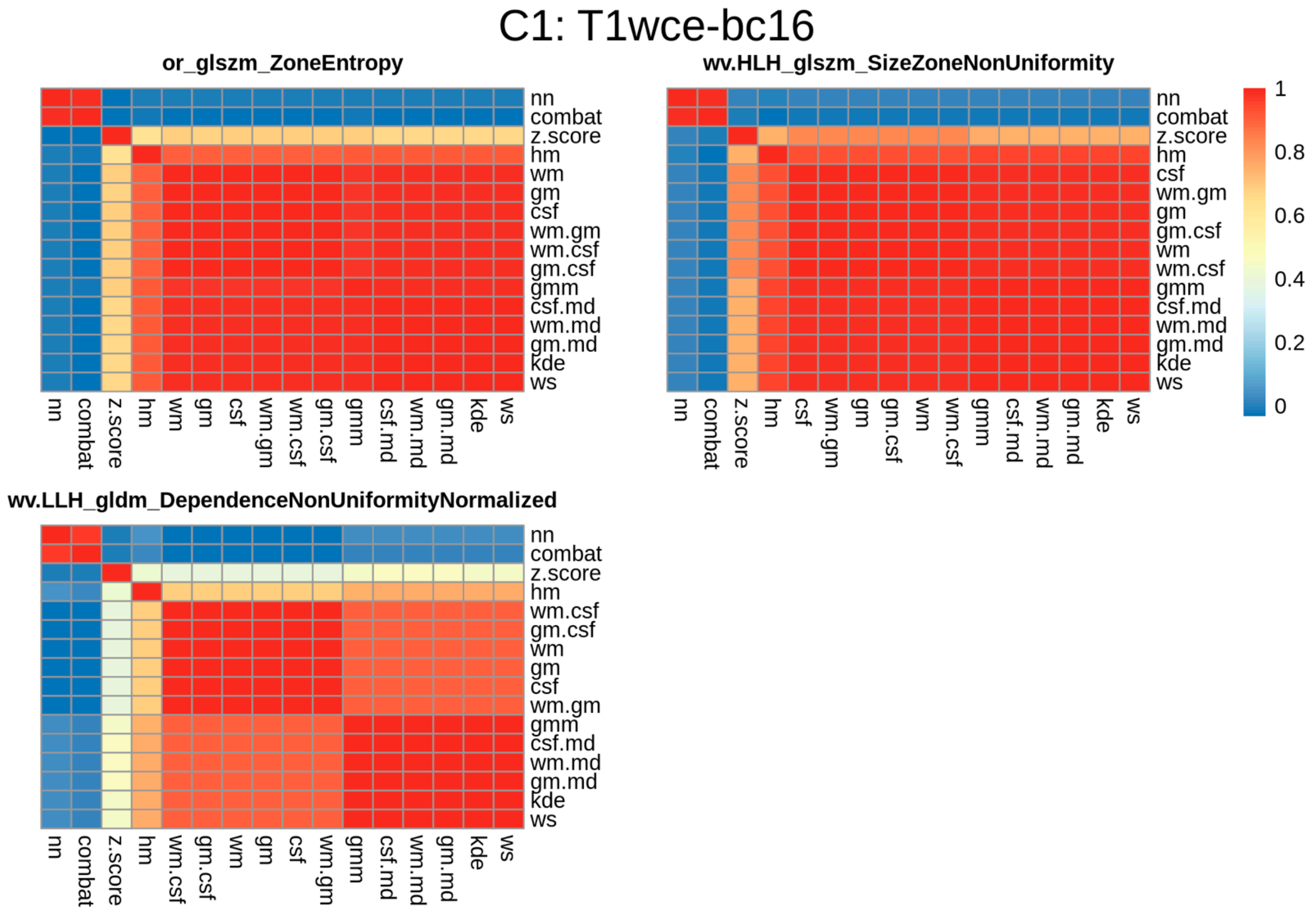

Supplementary Figure S1-6. Correlation heatmaps between the 15 different normalization methods considered and the reference non-normalized dataset for T1wce significant features in cohort C1 discretized with bin counts of 16.

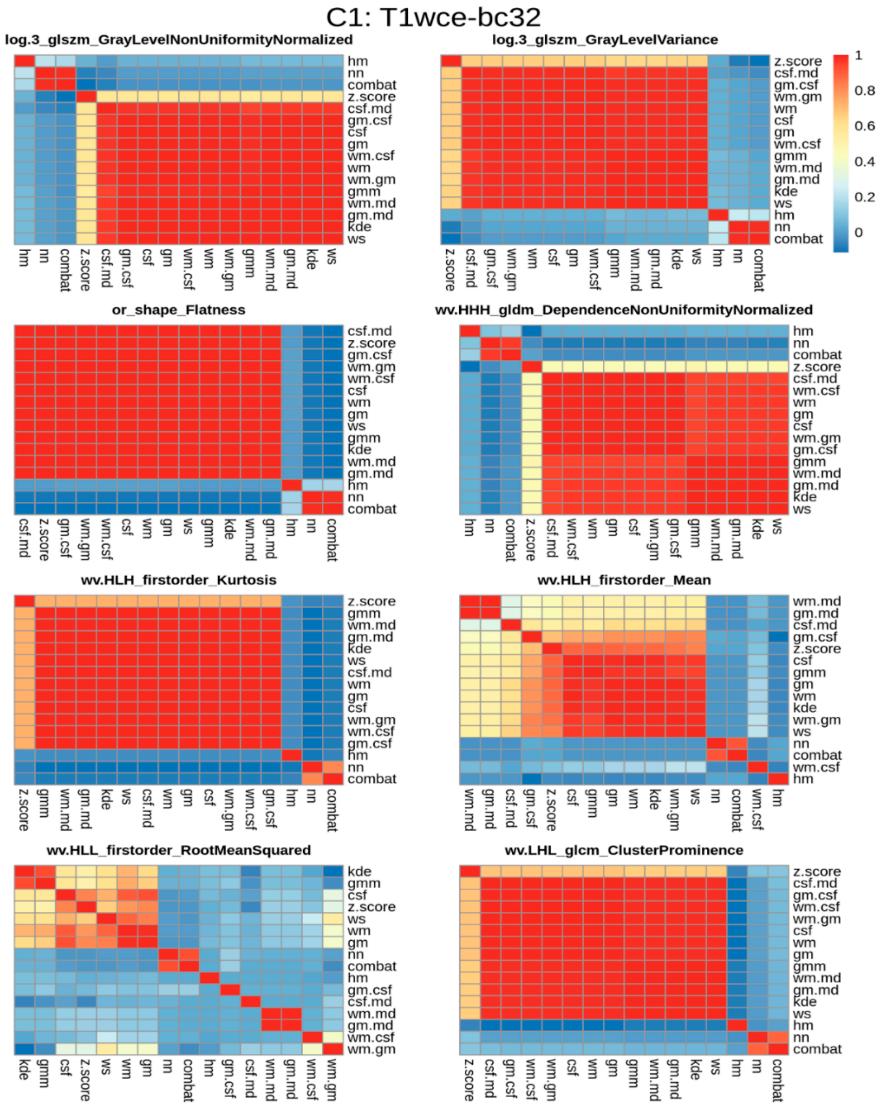

Supplementary Figure S1-7. Correlation heatmaps between the 15 different normalization methods considered and the reference non-normalized dataset for T1wce significant features in cohort C1 discretized with bin counts of 32.

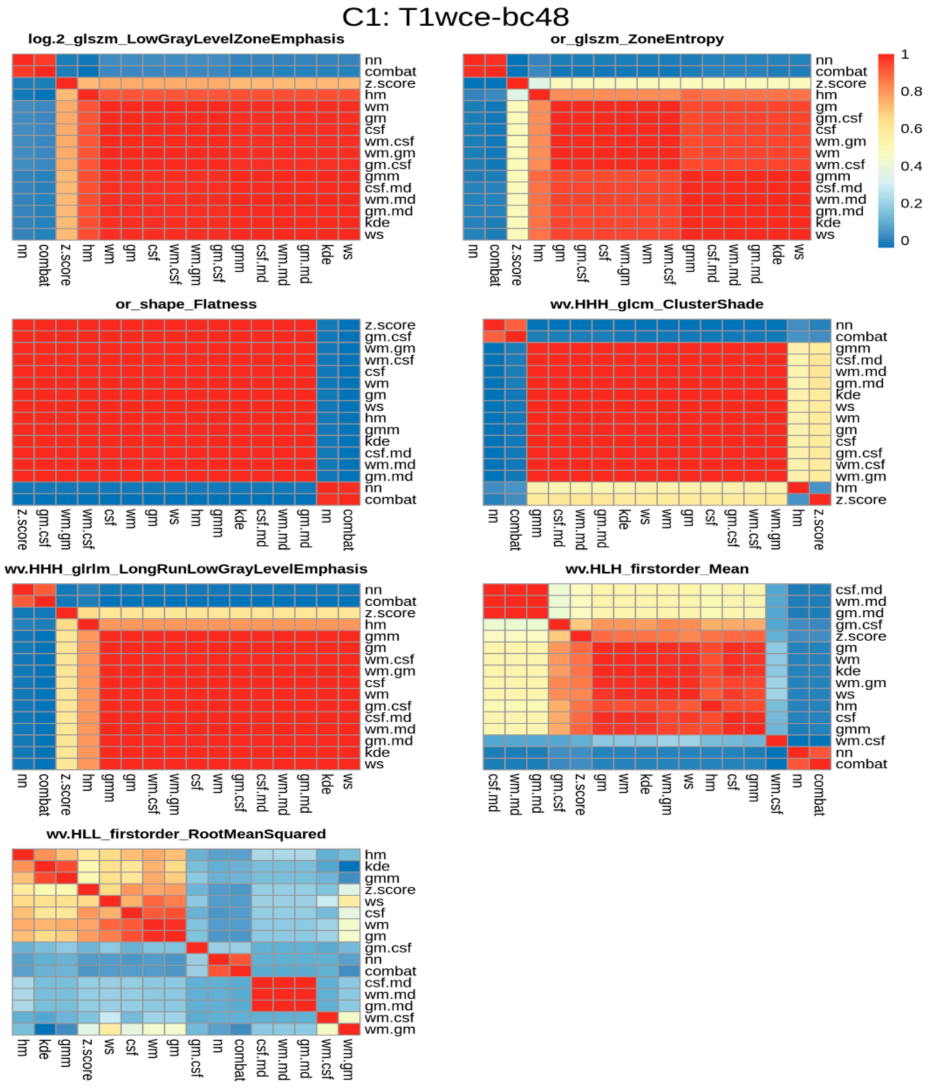

Supplementary Figure S1-8. Correlation heatmaps between the 15 different normalization methods considered and the reference non-normalized dataset for T1wce significant features in cohort C1 discretized with bin counts of 48.

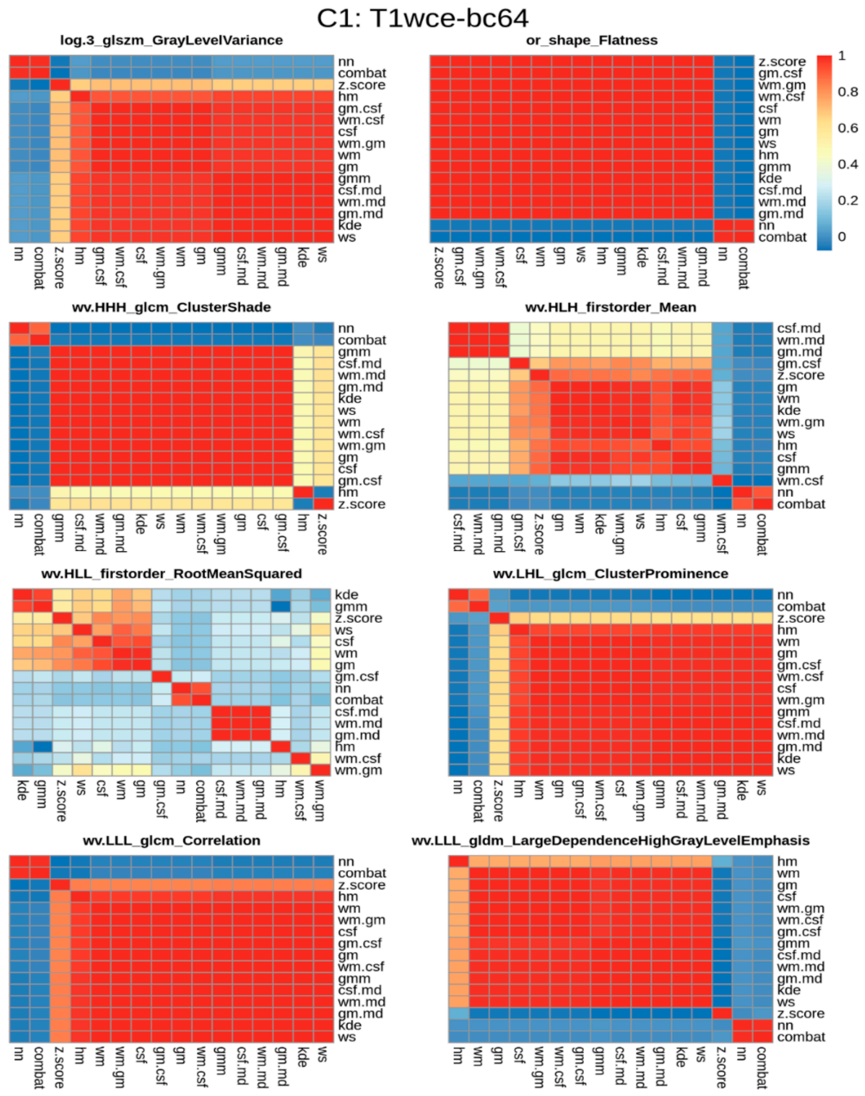

Supplementary Figure S1-9. Correlation heatmaps between the 15 different normalization methods considered and the reference non-normalized dataset for T1wce significant features in cohort C1 discretized with bin counts of 64.

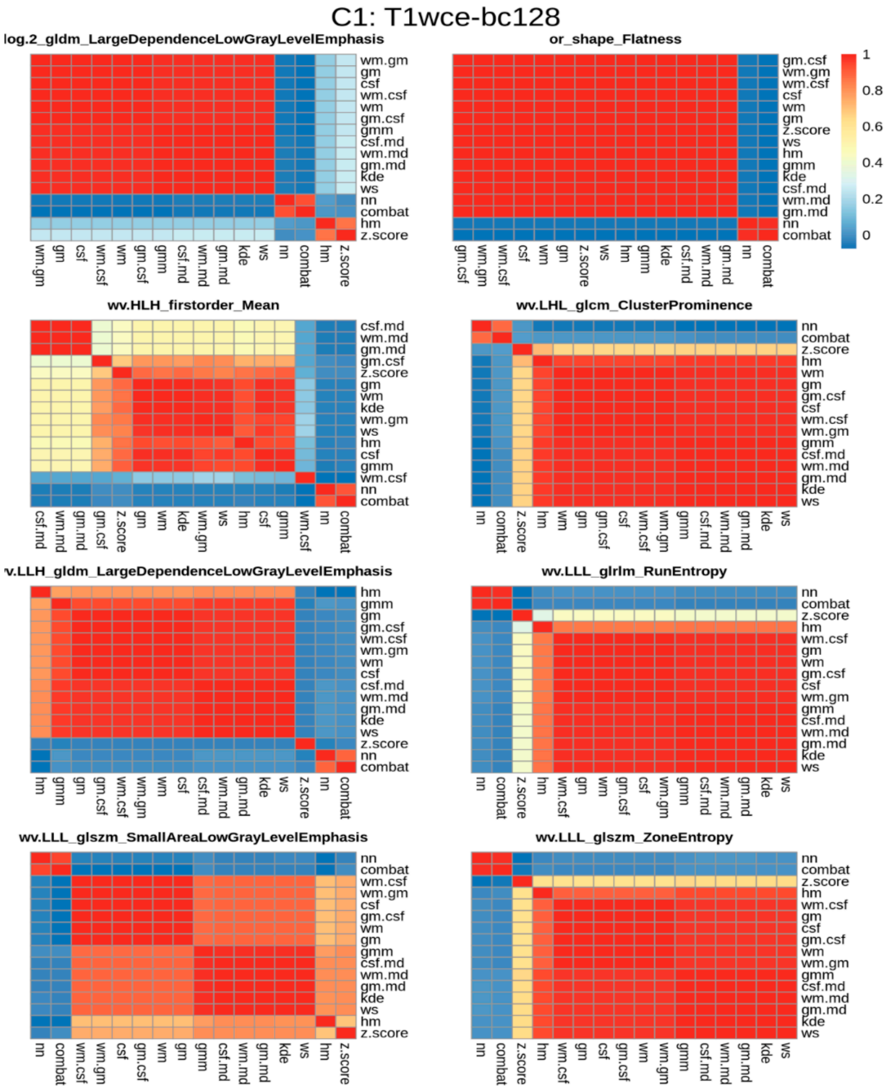

Supplementary Figure S1-10. Correlation heatmaps between the 15 different normalization methods considered and the reference non-normalized dataset for T1wce significant features in cohort C1 discretized with bin counts of 128.

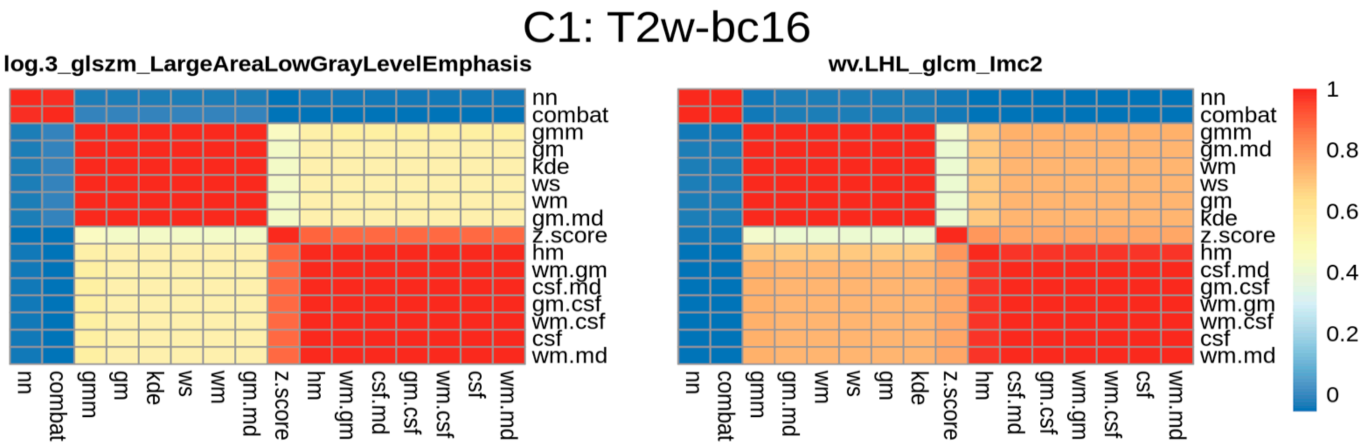

Supplementary Figure S1-11. Correlation heatmaps between the 15 different normalization methods considered and the reference non-normalized dataset for T2w significant features in cohort C1 discretized with bin counts of 16.

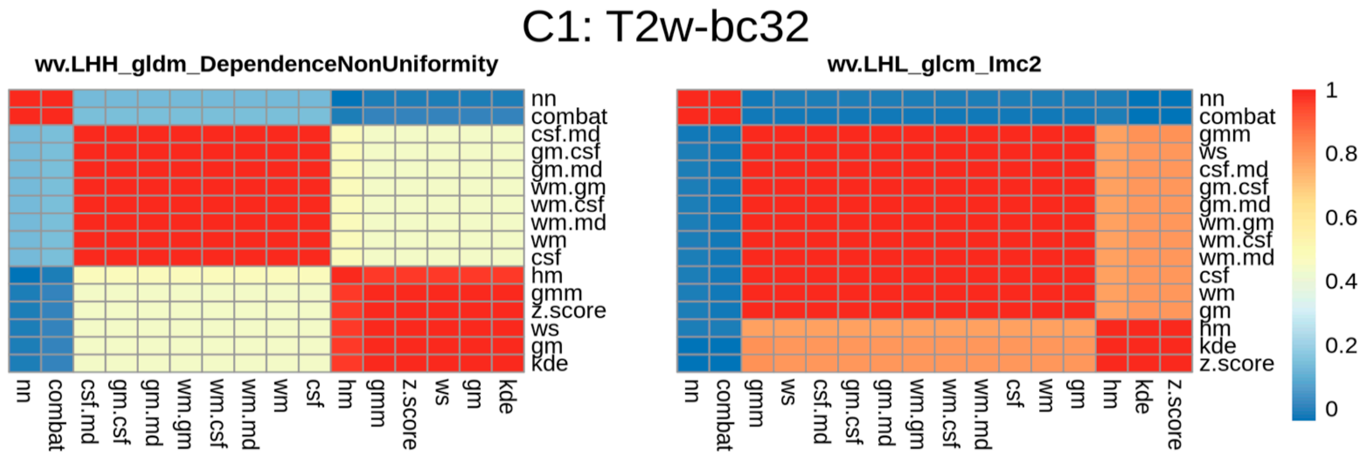

Supplementary Figure S1-12. Correlation heatmaps between the 15 different normalization methods considered and the reference non-normalized dataset for T2w significant features in cohort C1 discretized with bin counts of 32.

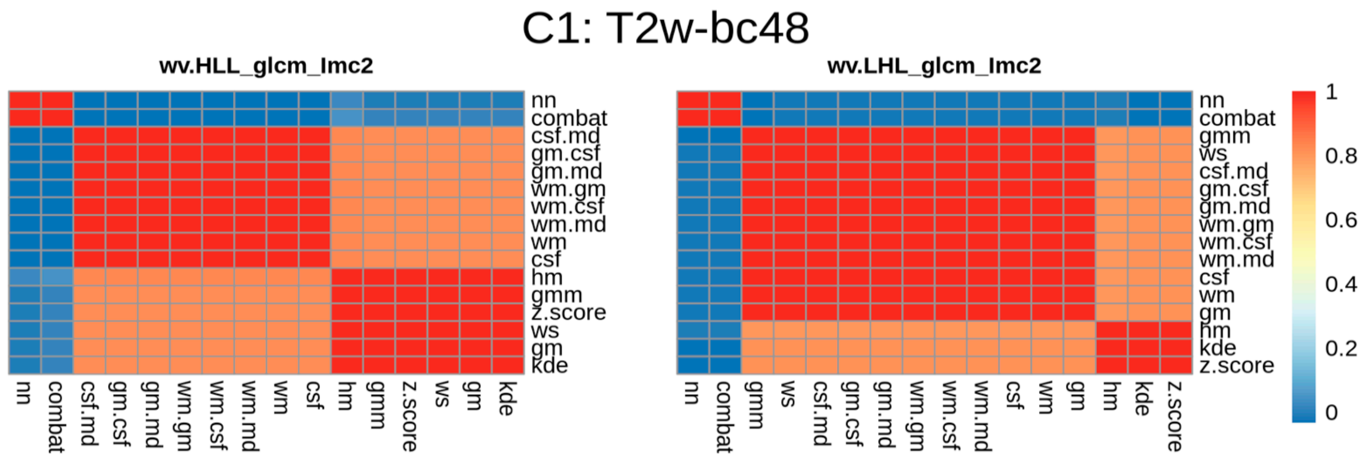

Supplementary Figure S1-13. Correlation heatmaps between the 15 different normalization methods considered and the reference non-normalized dataset for T2w significant features in cohort C1 discretized with bin counts of 48.

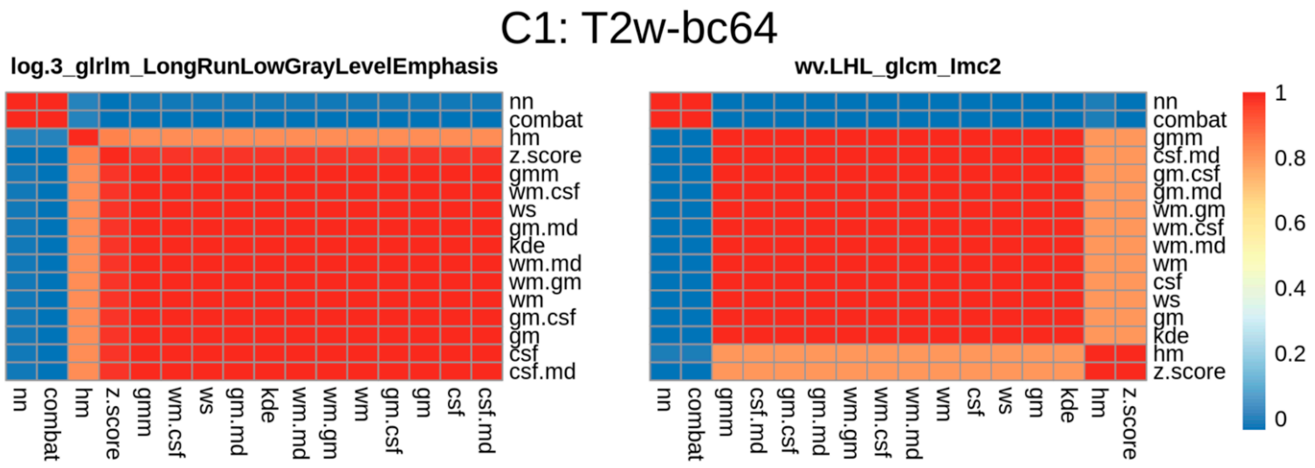

Supplementary Figure S1-14. Correlation heatmaps between the 15 different normalization methods considered and the reference non-normalized dataset for T2w significant features in cohort C1 discretized with bin counts of 64.

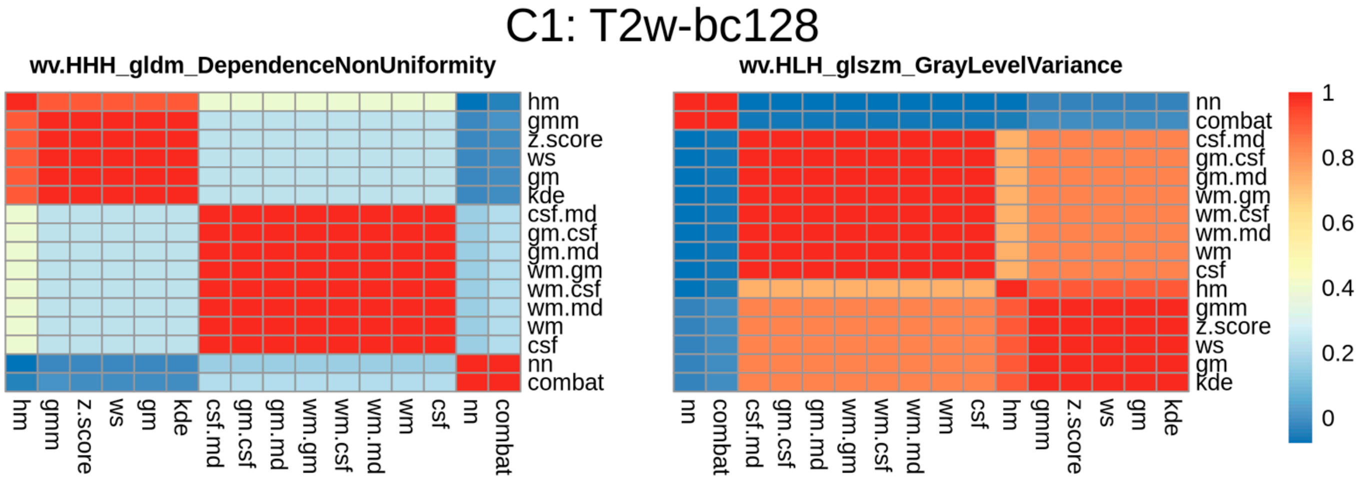

Supplementary Figure S1-15. Correlation heatmaps between the 15 different normalization methods considered and the reference non-normalized dataset for T2w significant features in cohort C1 discretized with bin counts of 128.

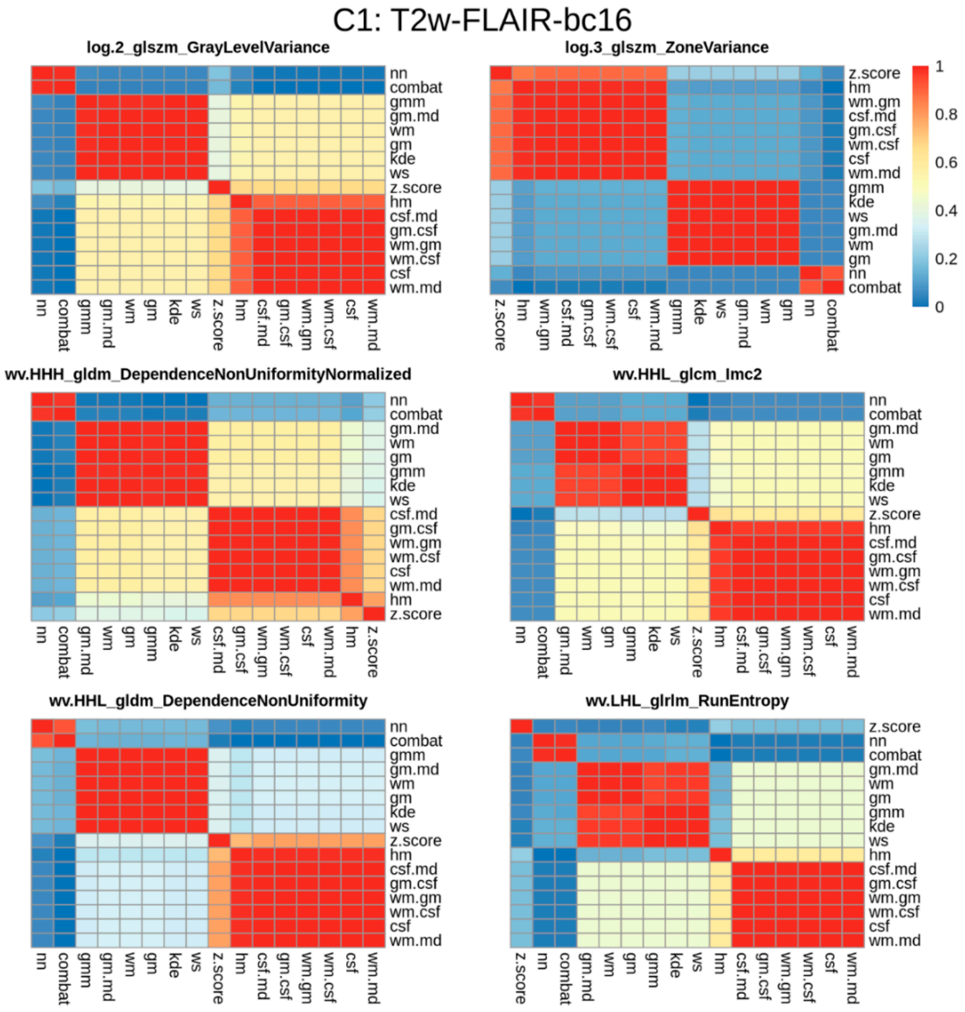

Supplementary Figure S1-16. Correlation heatmaps between the 15 different normalization methods considered and the reference non-normalized dataset for T2w-FLAIR significant features in cohort C1 discretized with bin counts of 16.

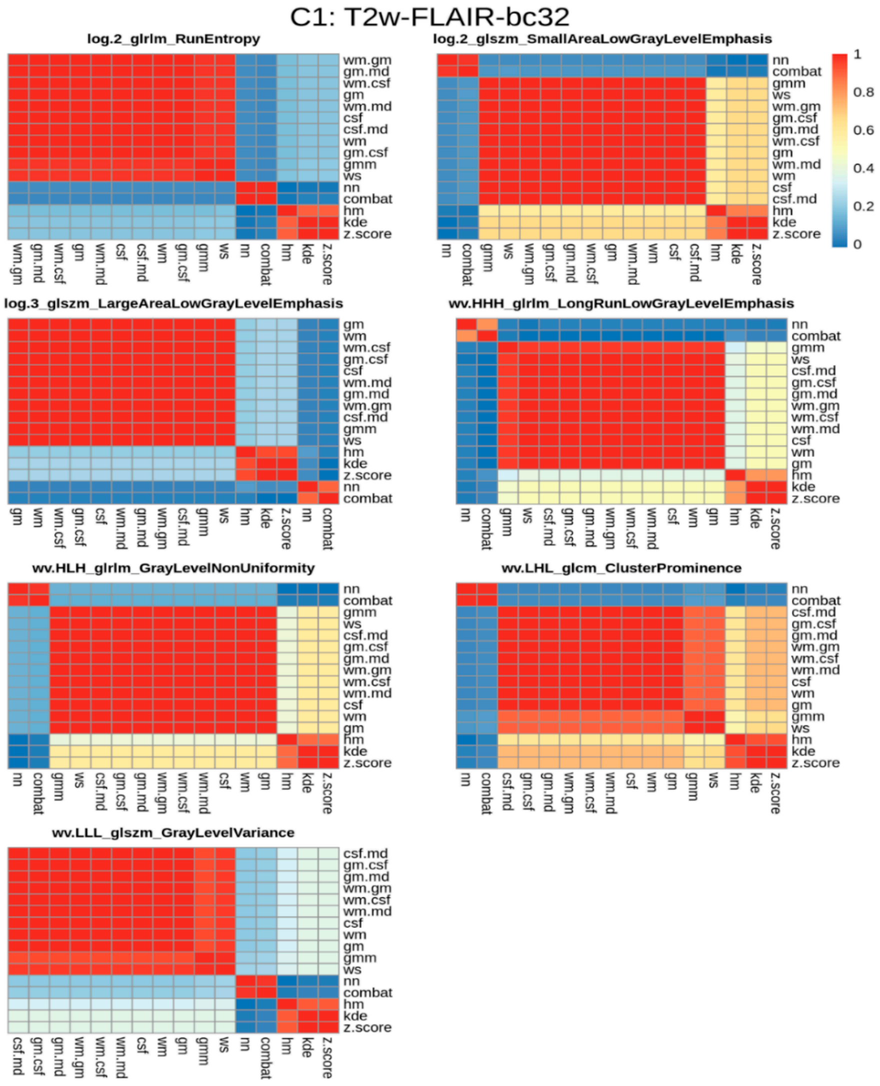

Supplementary Figure S1-17. Correlation heatmaps between the 15 different normalization methods considered and the reference non-normalized dataset for T2w-FLAIR significant features in cohort C1 discretized with bin counts of 32.

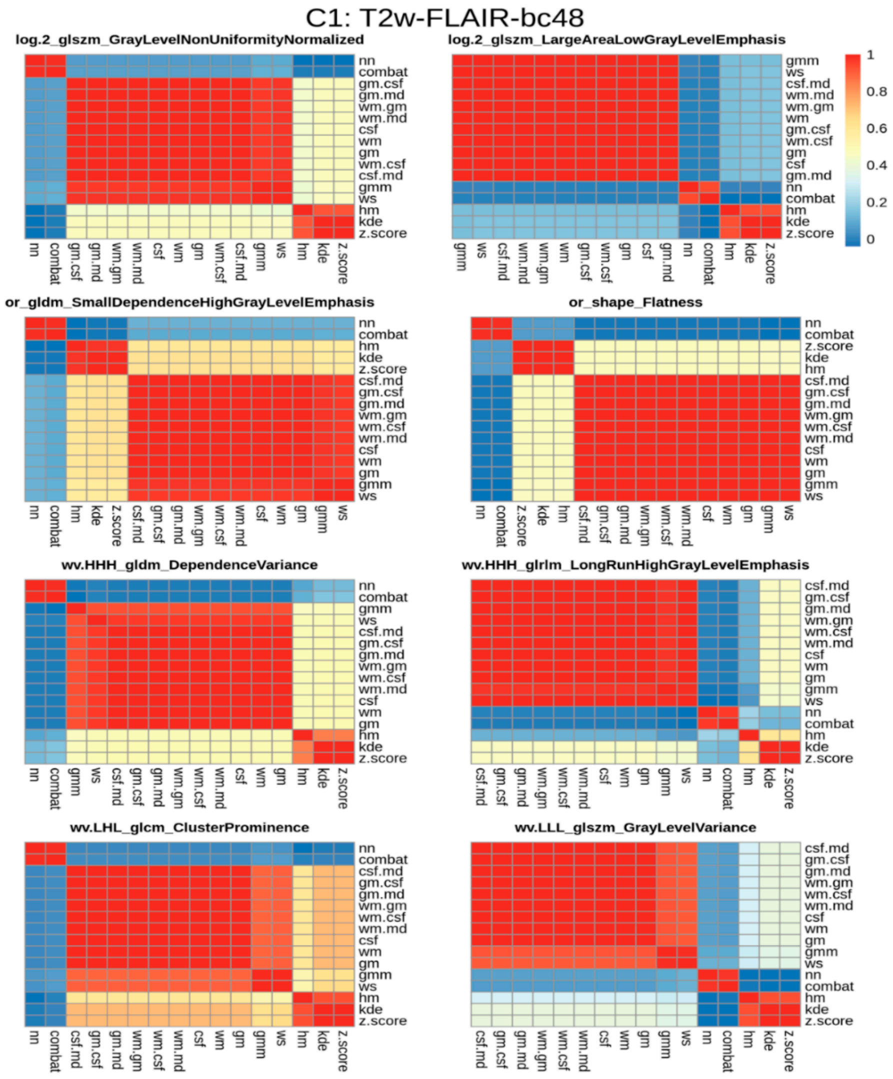

Supplementary Figure S1-18. Correlation heatmaps between the 15 different normalization methods considered and the reference non-normalized dataset for T2w-FLAIR significant features in cohort C1 discretized with bin counts of 48.

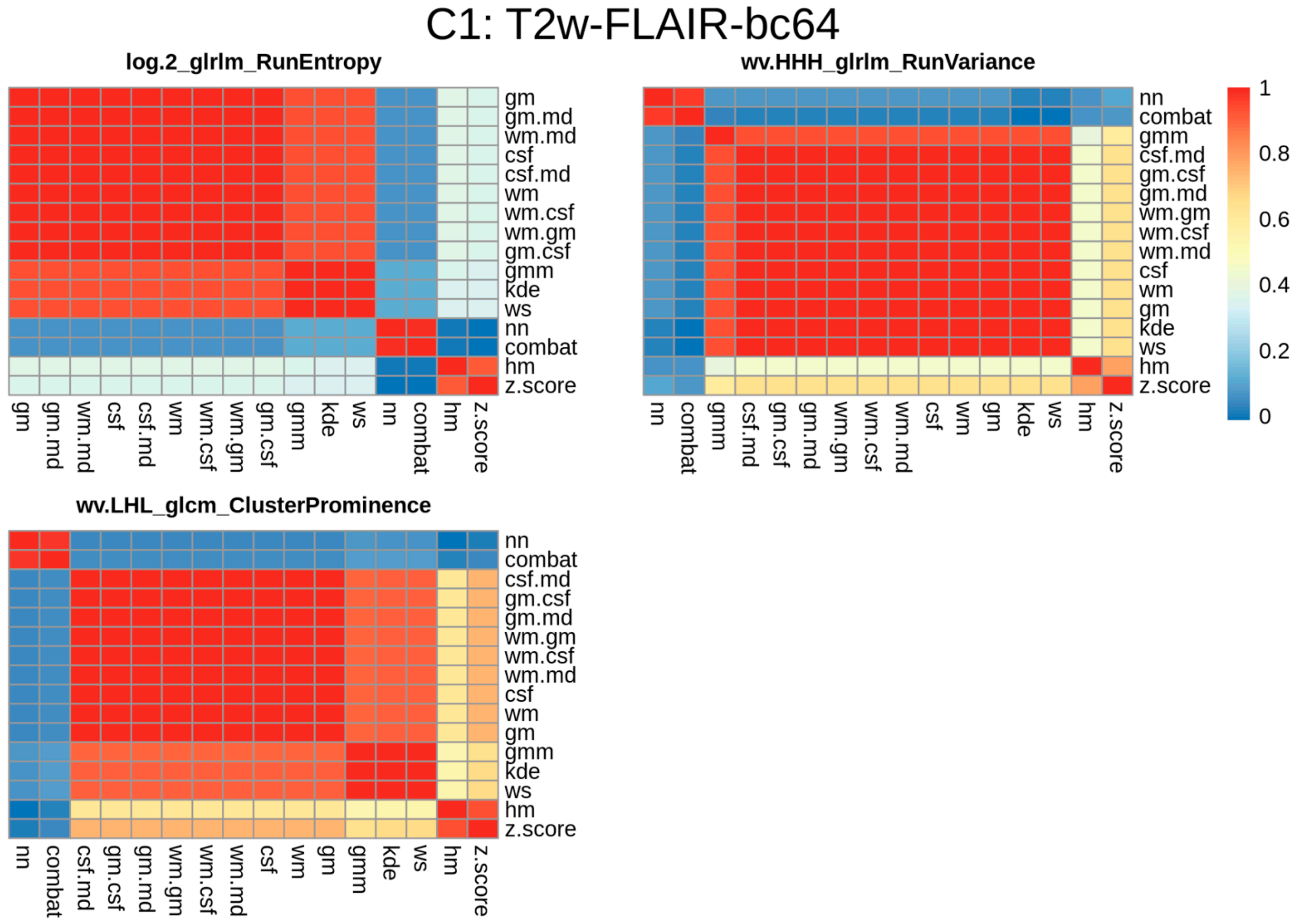

Supplementary Figure S1-19. Correlation heatmaps between the 15 different normalization methods considered and the reference non-normalized dataset for T2w-FLAIR significant features in cohort C1 discretized with bin counts of 64.

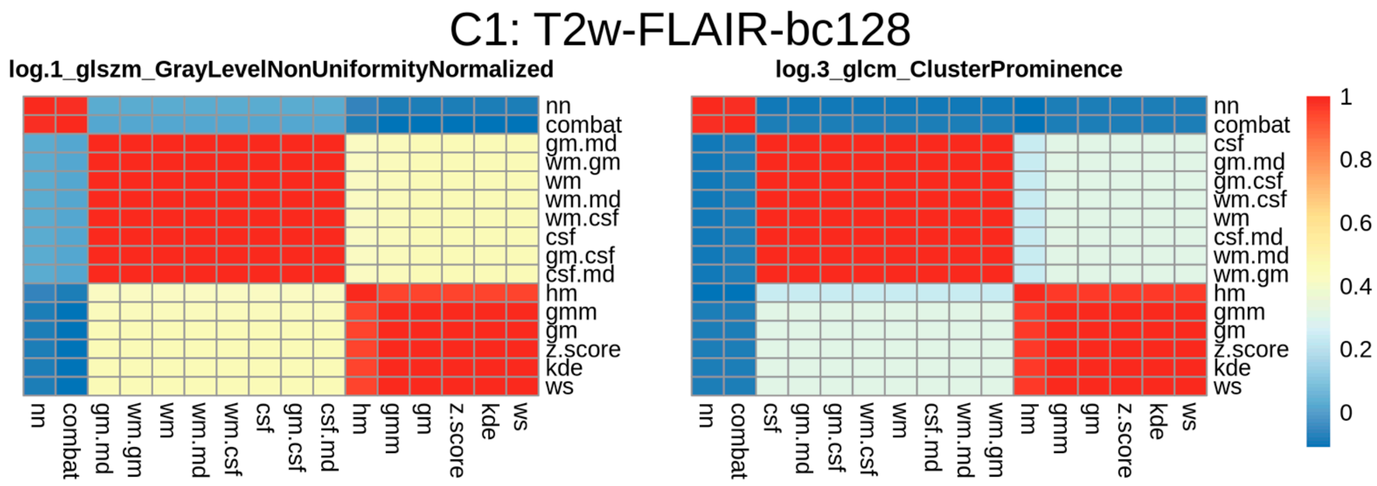

Supplementary Figure S1-20. Correlation heatmaps between the 15 different normalization methods considered and the reference non-normalized dataset for T2w-FLAIR significant features in cohort C1 discretized with bin counts of 128.

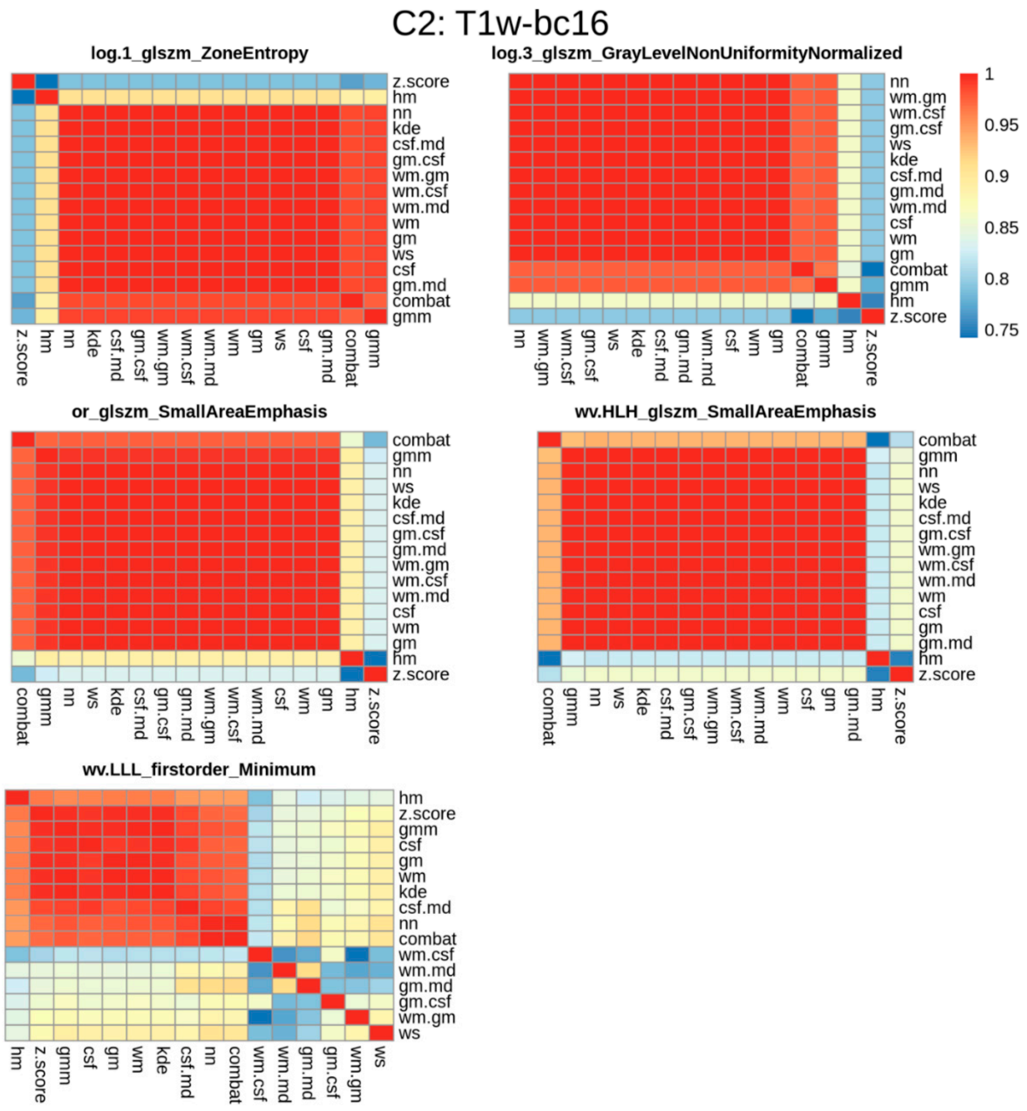

Supplementary Figure S1-21. Correlation heatmaps between the 15 different normalization methods considered and the reference non-normalized dataset for T1w significant features in cohort C2 discretized with bin counts of 16.

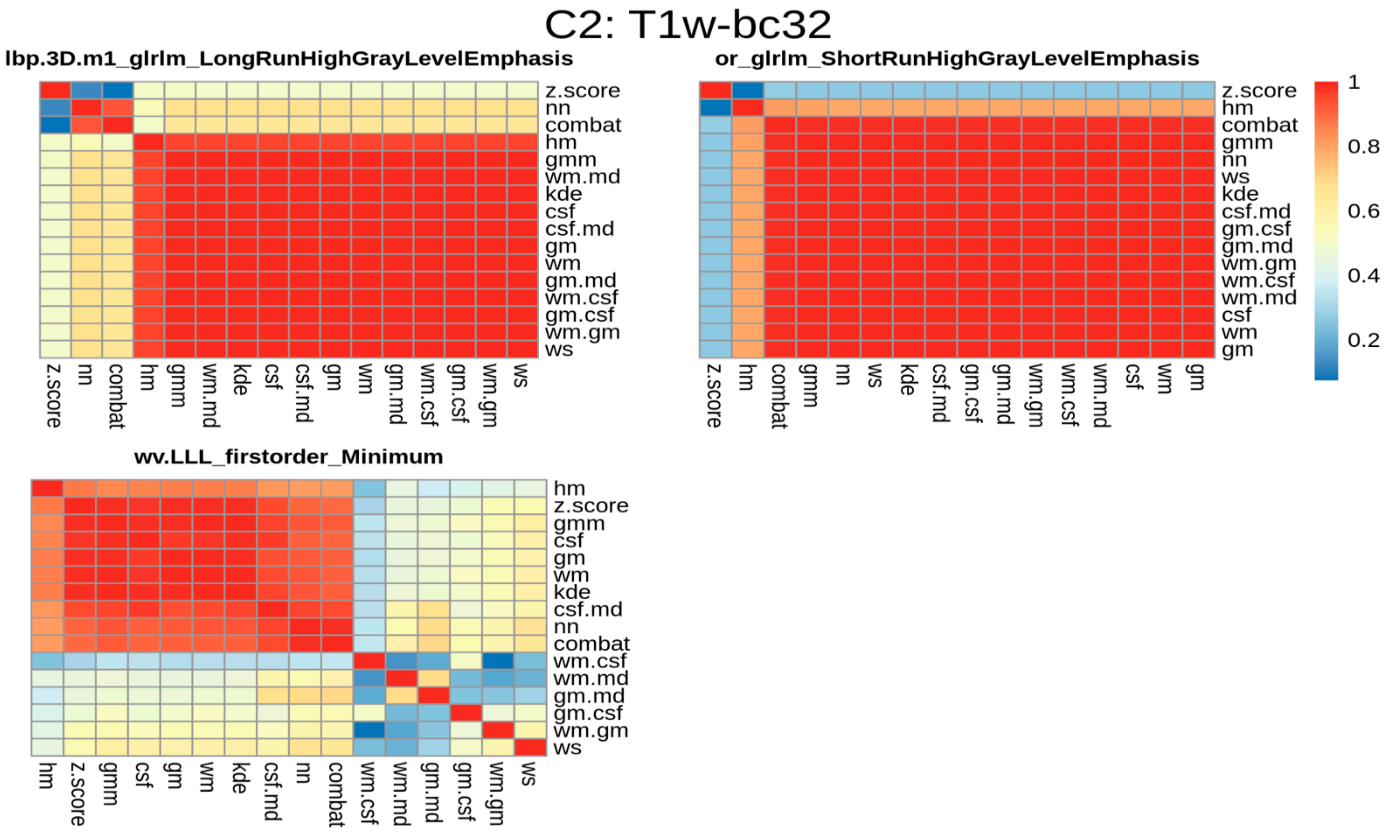

Supplementary Figure S1-22. Correlation heatmaps between the 15 different normalization methods considered and the reference non-normalized dataset for T1w significant features in cohort C2 discretized with bin counts of 32.

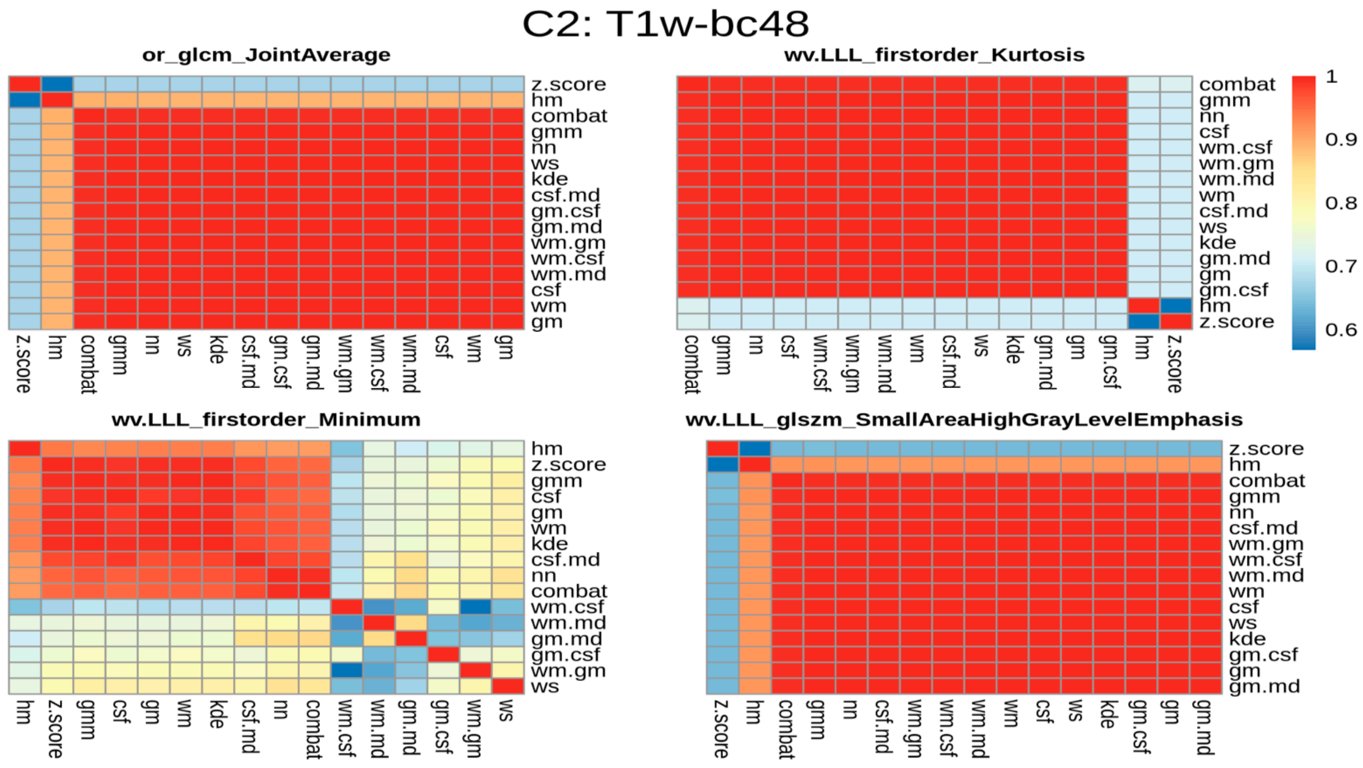

Supplementary Figure S1-23. Correlation heatmaps between the 15 different normalization methods considered and the reference non-normalized dataset for T1w significant features in cohort C2 discretized with bin counts of 48.

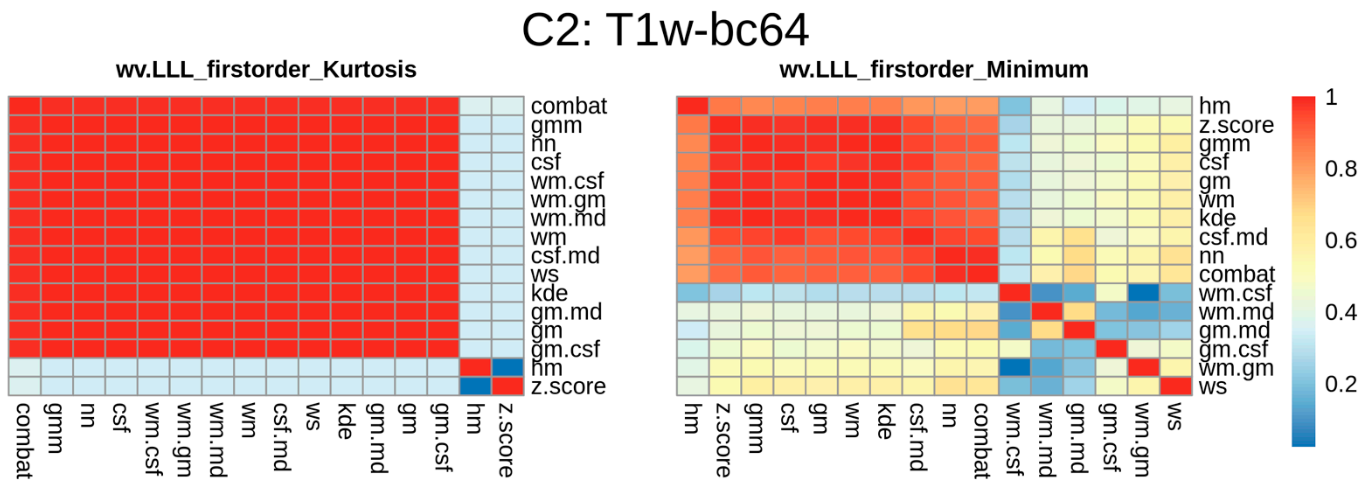

Supplementary Figure S1-24. Correlation heatmaps between the 15 different normalization methods considered and the reference non-normalized dataset for T1w significant features in cohort C2 discretized with bin counts of 64.

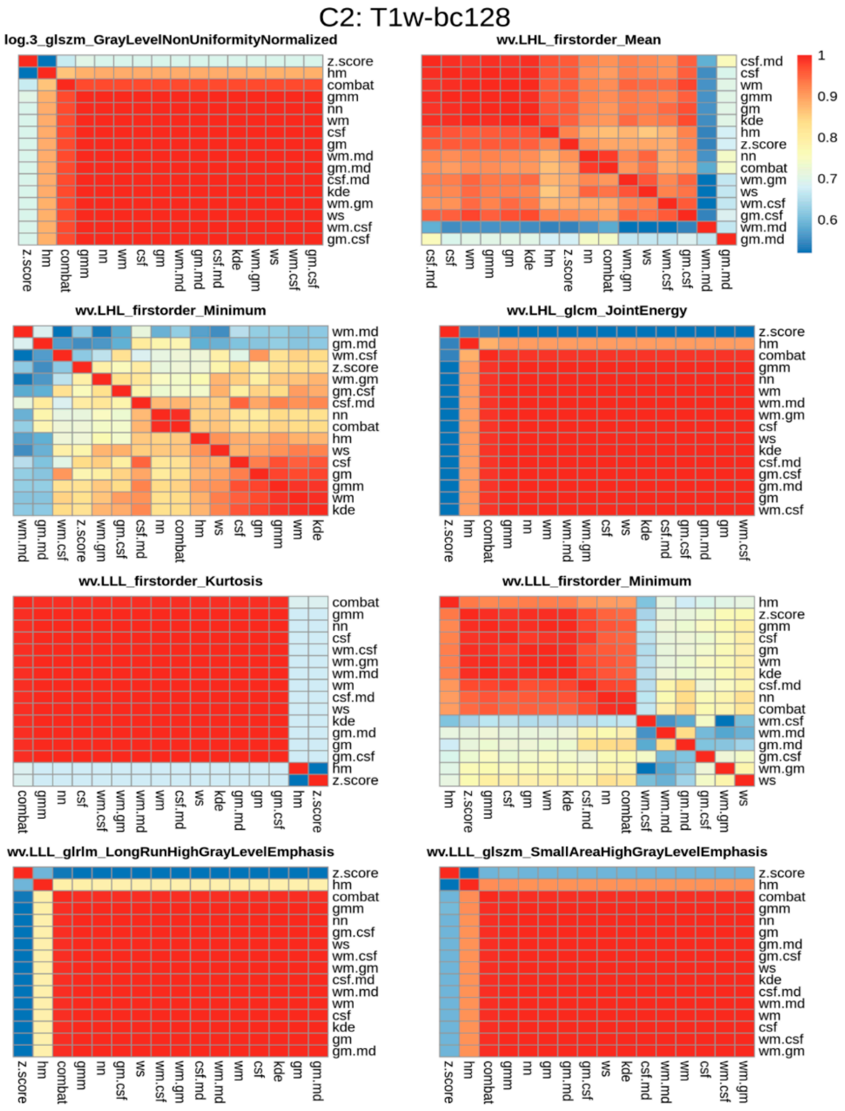

Supplementary Figure S1-25. Correlation heatmaps between the 15 different normalization methods considered and the reference non-normalized dataset for T1w significant features in cohort C2 discretized with bin counts of 128.

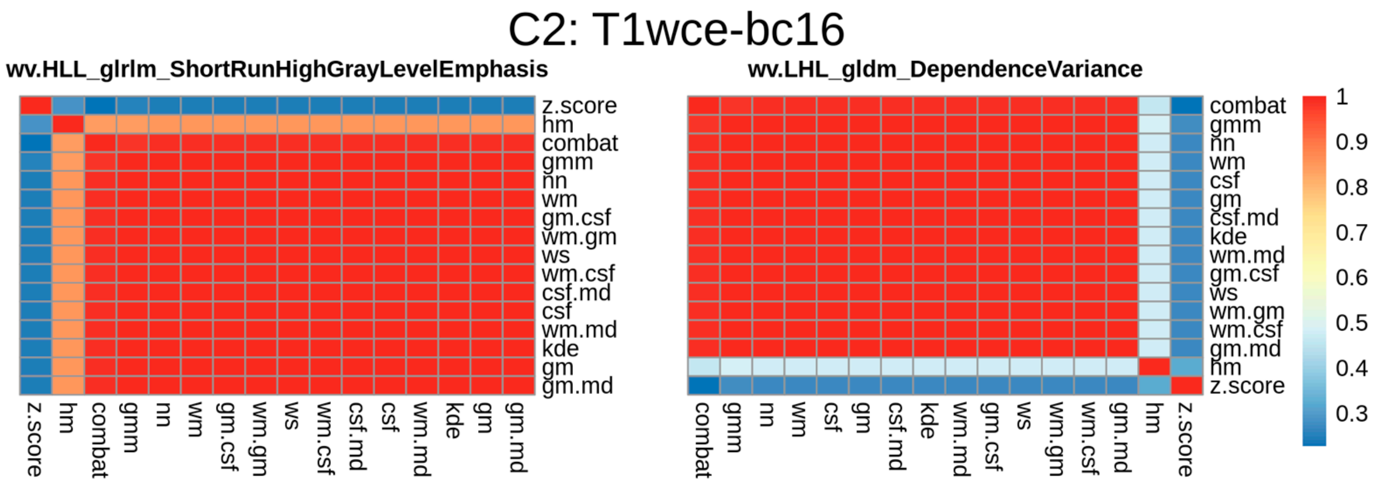

Supplementary Figure S1-26. Correlation heatmaps between the 15 different normalization methods considered and the reference non-normalized dataset for T1wce significant features in cohort C2 discretized with bin counts of 16.

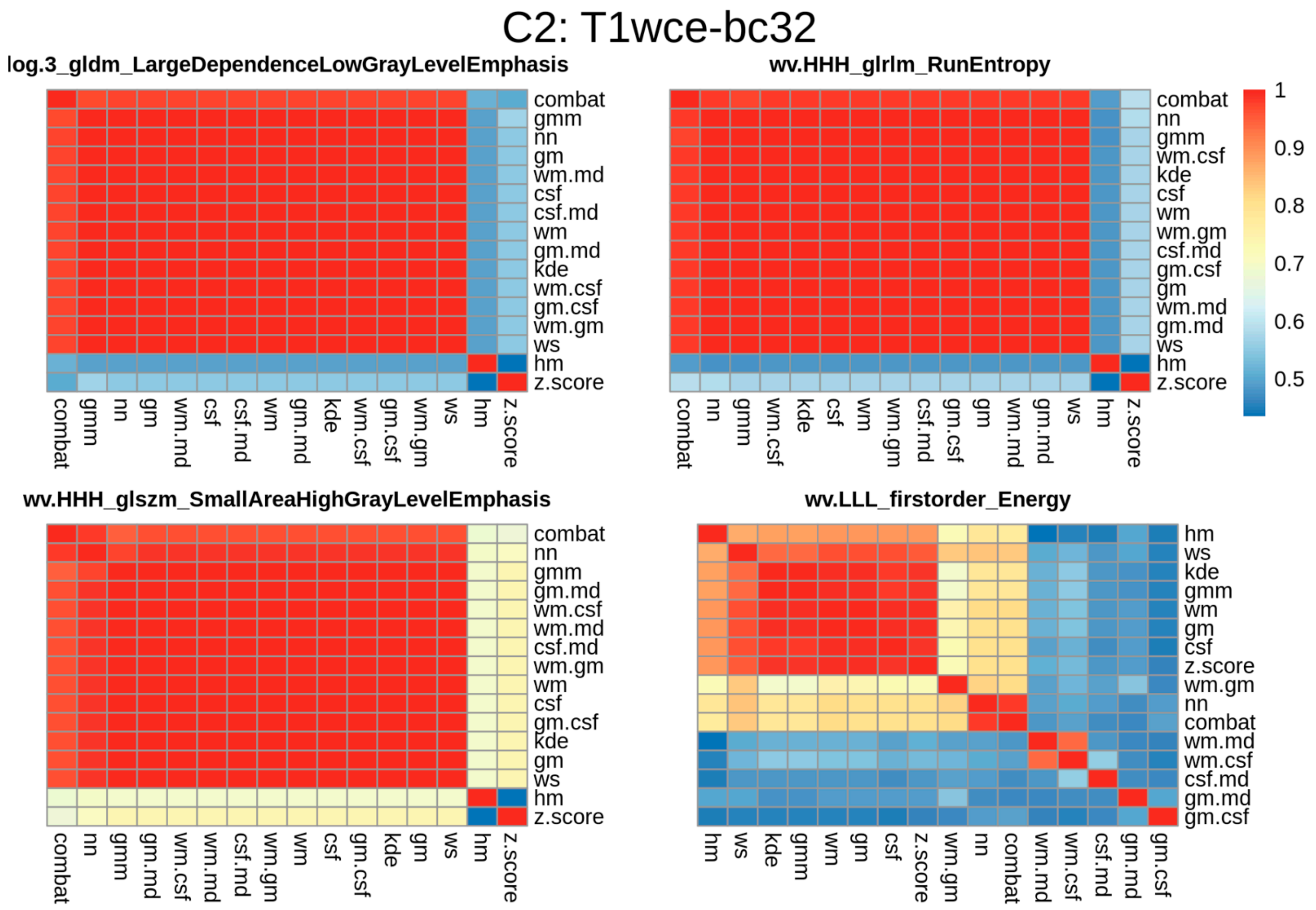

Supplementary Figure S1-27. Correlation heatmaps between the 15 different normalization methods considered and the reference non-normalized dataset for T1wce significant features in cohort C2 discretized with bin counts of 32.

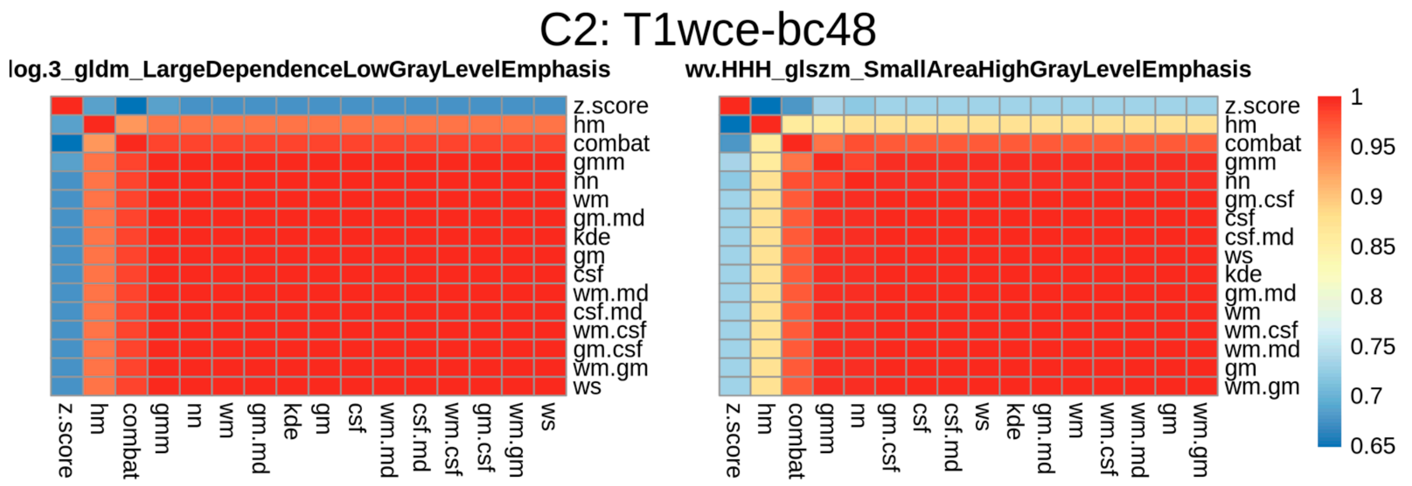

Supplementary Figure S1-28. Correlation heatmaps between the 15 different normalization methods considered and the reference non-normalized dataset for T1wce significant features in cohort C2 discretized with bin counts of 48.

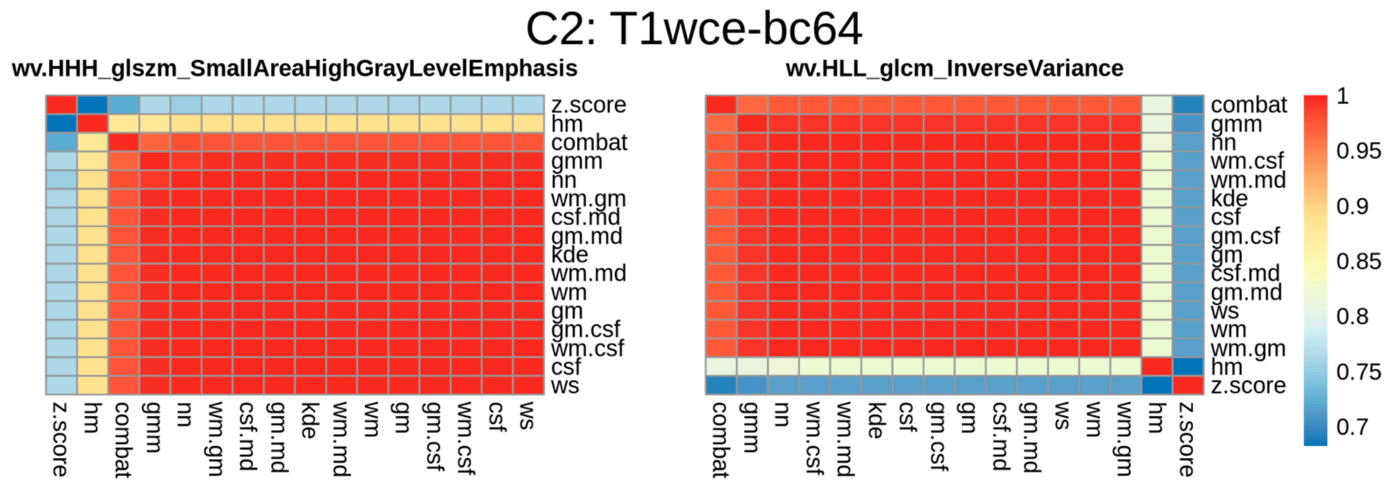

Supplementary Figure S1-29. Correlation heatmaps between the 15 different normalization methods considered and the reference non-normalized dataset for T1wce significant features in cohort C2 discretized with bin counts of 64.

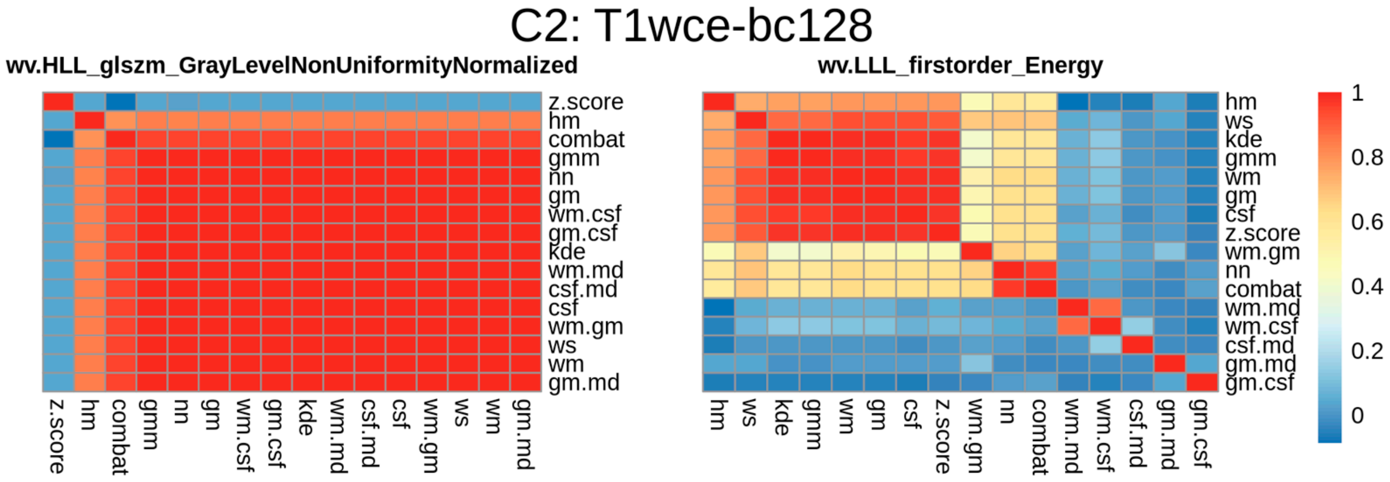

Supplementary Figure S1-30. Correlation heatmaps between the 15 different normalization methods considered and the reference non-normalized dataset for T1wce significant features in cohort C2 discretized with bin counts of 128.

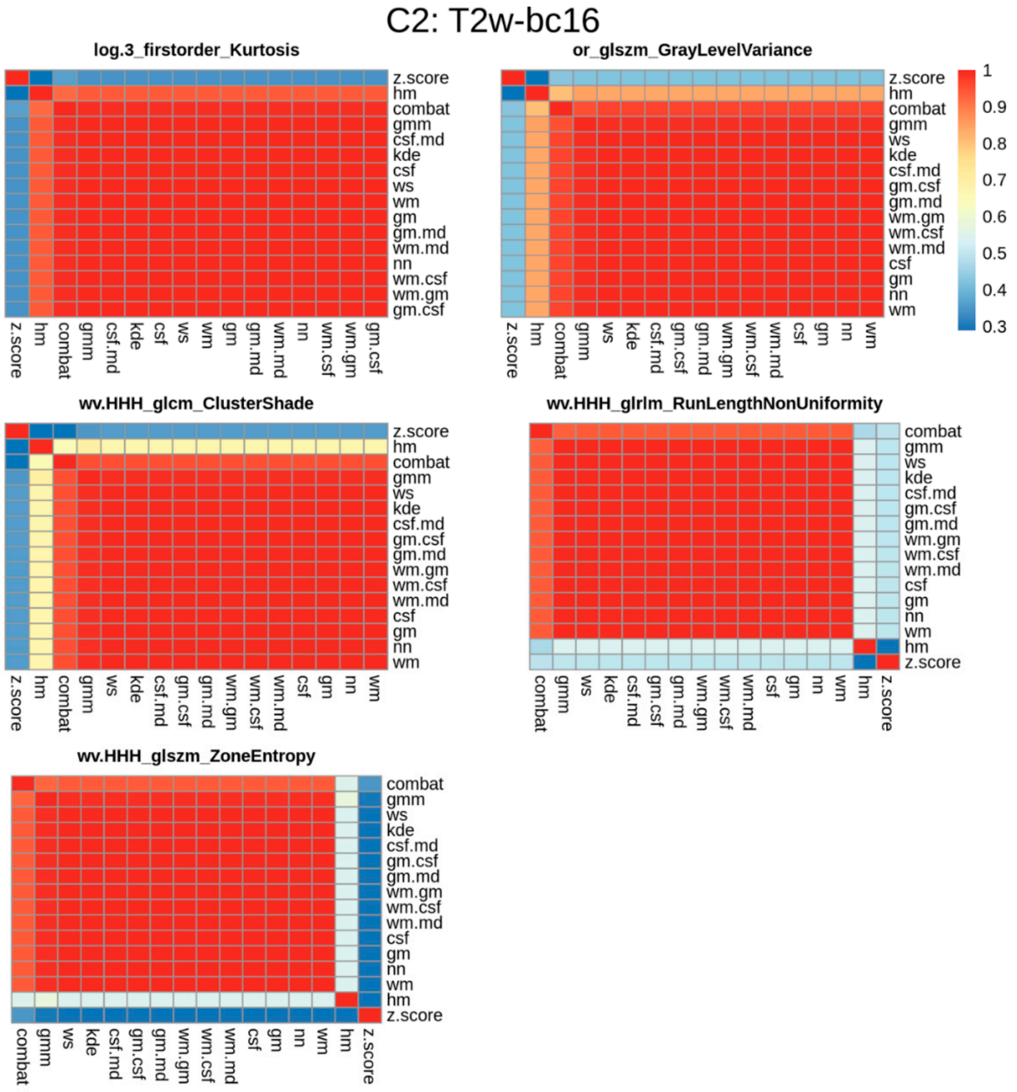

Supplementary Figure S1-31. Correlation heatmaps between the 15 different normalization methods considered and the reference non-normalized dataset for T2w significant features in cohort C2 discretized with bin counts of 16.

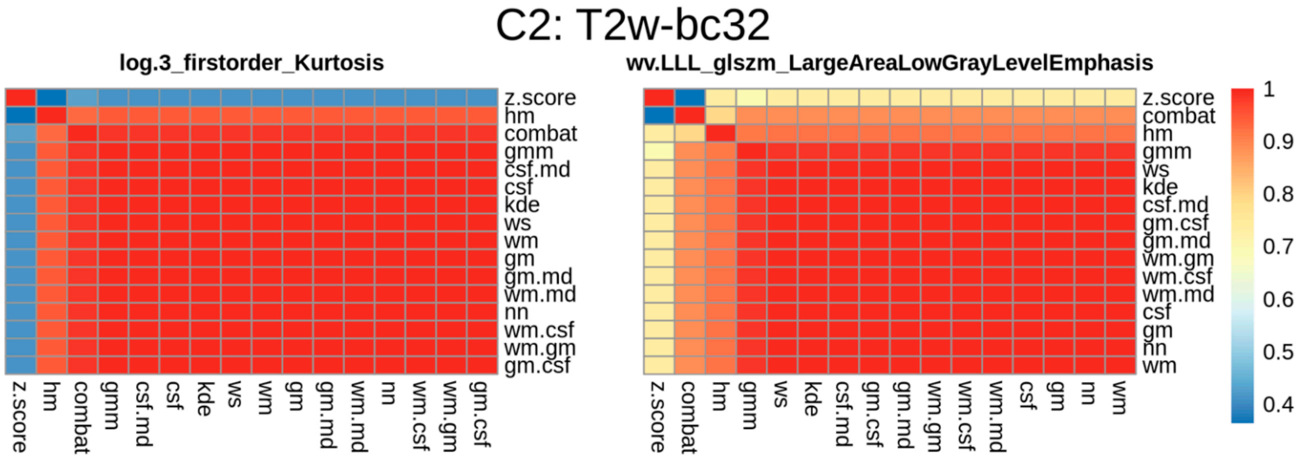

Supplementary Figure S1-32. Correlation heatmaps between the 15 different normalization methods considered and the reference non-normalized dataset for T2w significant features in cohort C2 discretized with bin counts of 32.

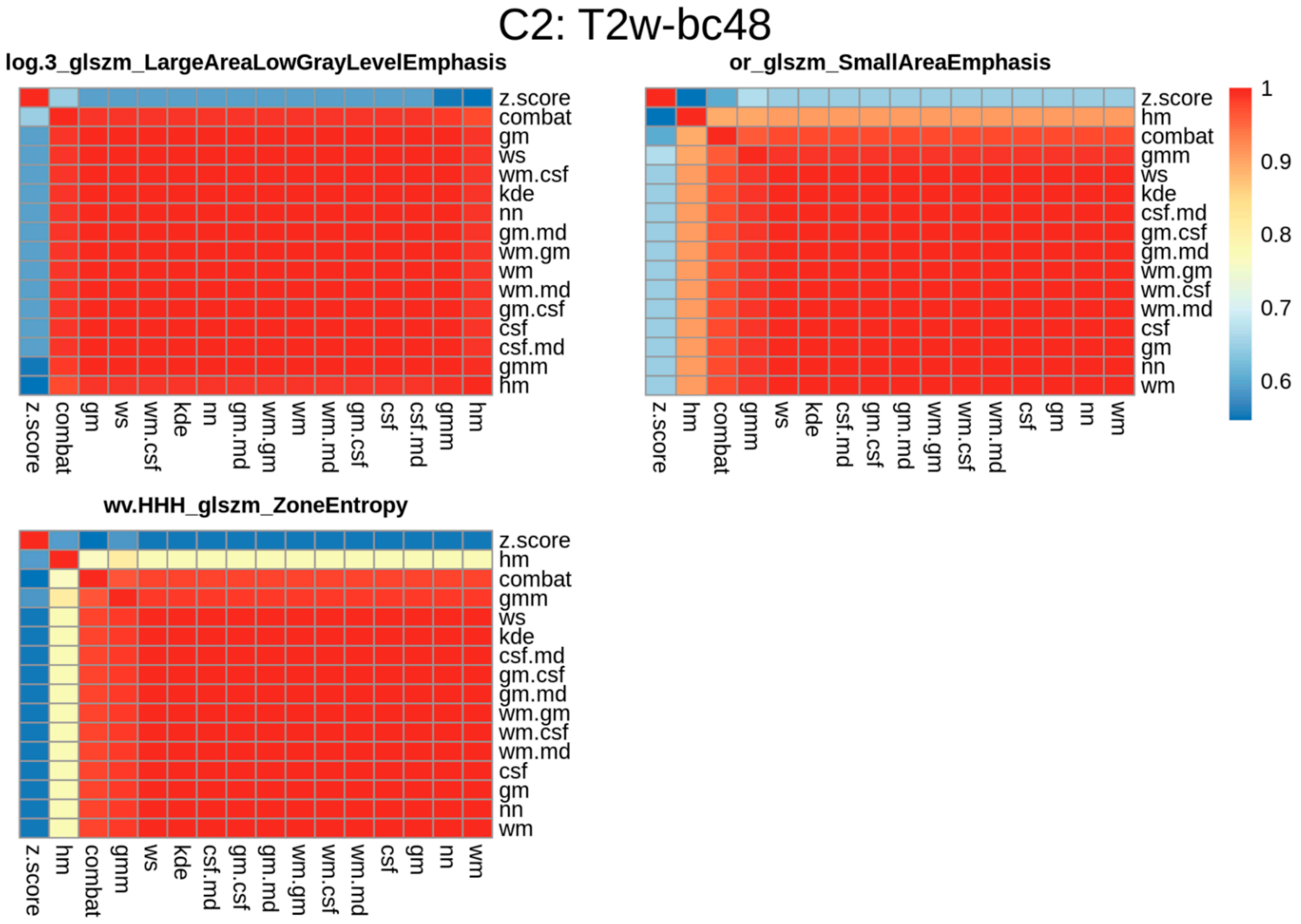

Supplementary Figure S1-33. Correlation heatmaps between the 15 different normalization methods considered and the reference non-normalized dataset for T2w significant features in cohort C2 discretized with bin counts of 48.

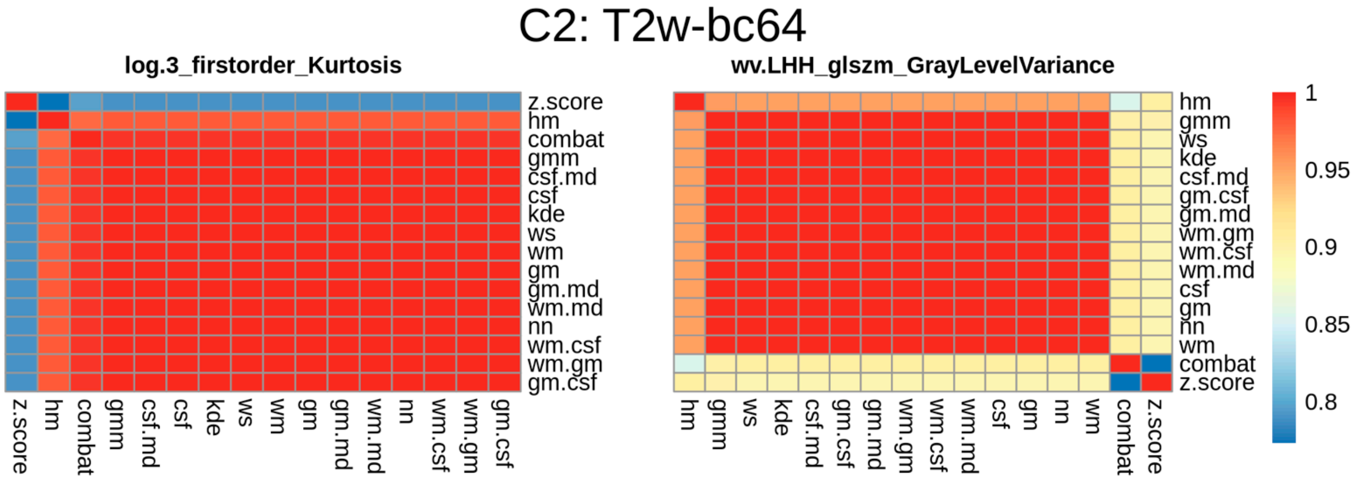

Supplementary Figure S1-34 Correlation heatmaps between the 15 different normalization methods considered and the reference non-normalized dataset for T2w significant features in cohort C2 discretized with bin counts of 64.

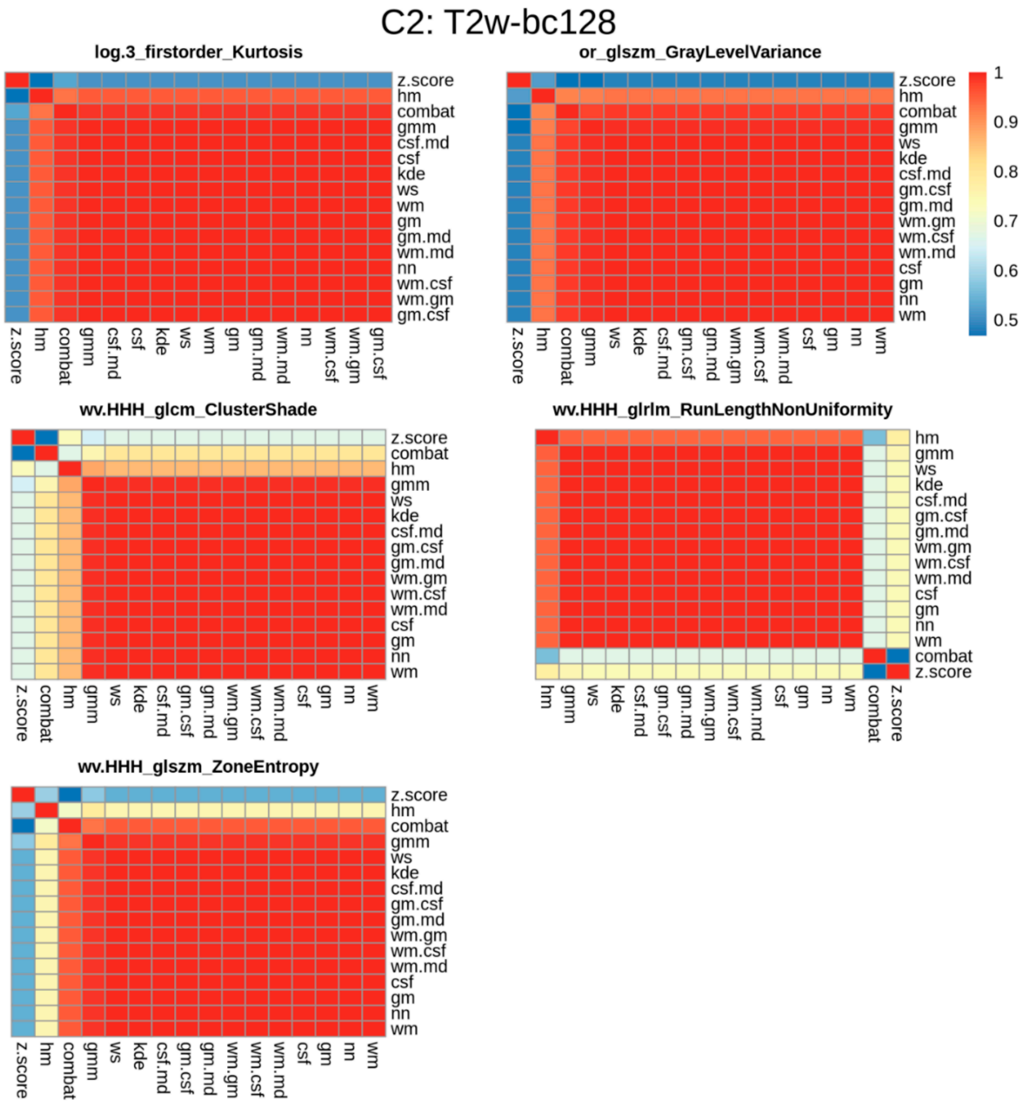

Supplementary Figure S1-35. Correlation heatmaps between the 15 different normalization methods considered and the reference non-normalized dataset for T2w significant features in cohort C2 discretized with bin counts of 128.

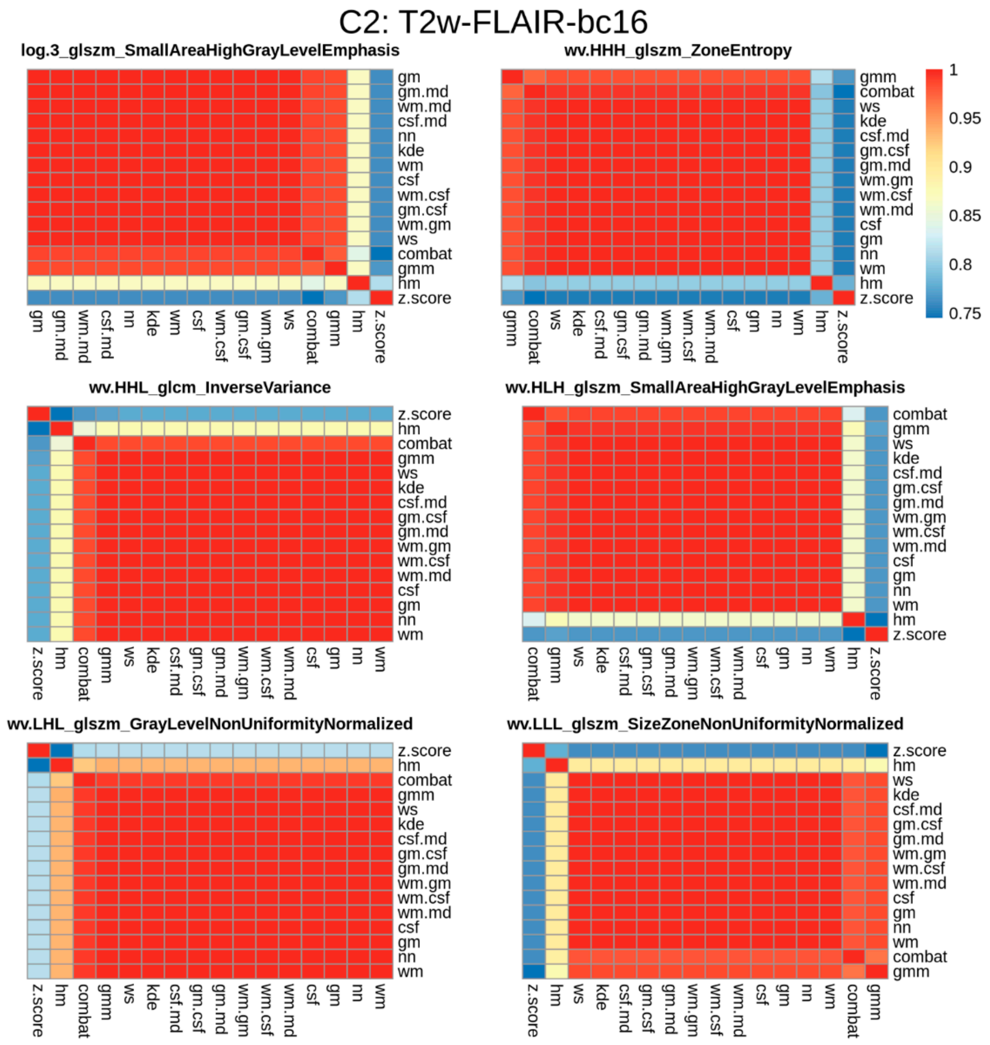

Supplementary Figure S1-36. Correlation heatmaps between the 15 different normalization methods considered and the reference non-normalized dataset for T2w-FLAIR significant features in cohort C2 discretized with bin counts of 16.

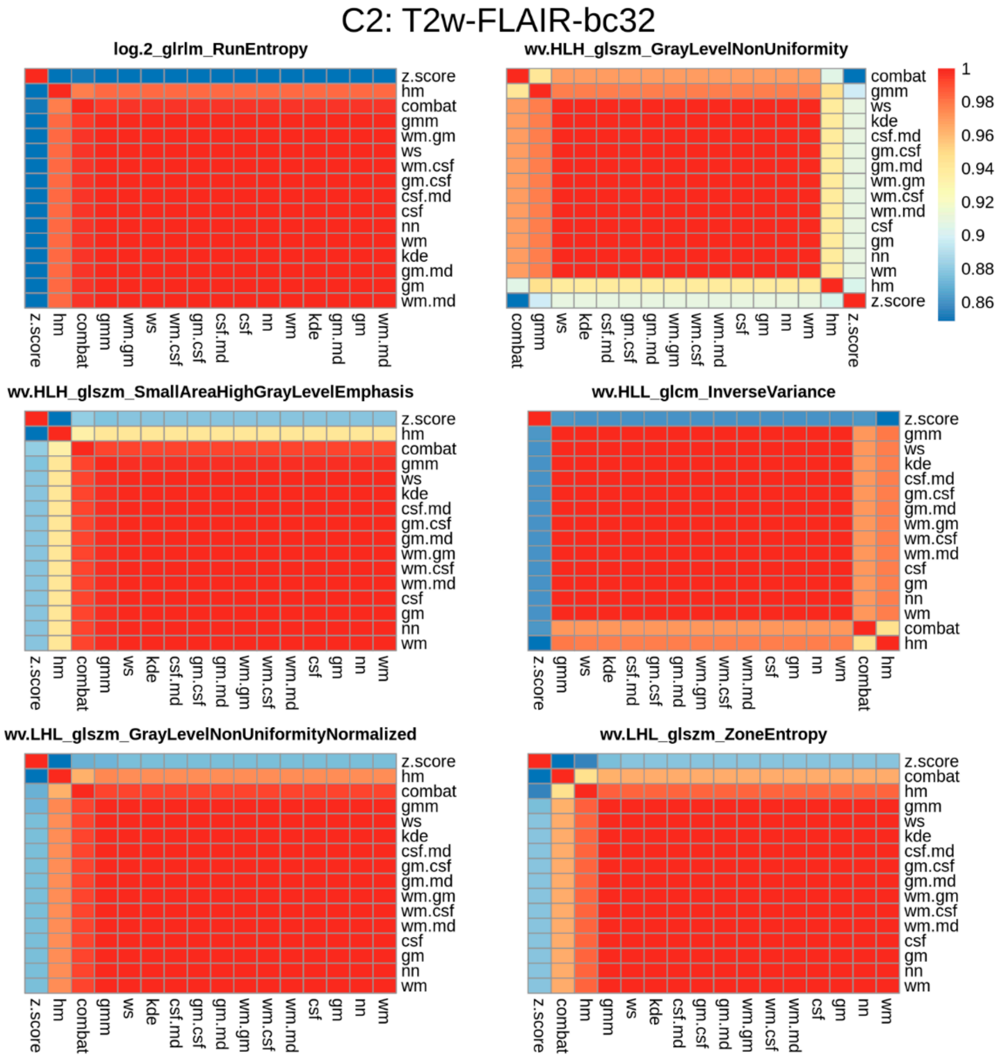

Supplementary Figure S1-37. Correlation heatmaps between the 15 different normalization methods considered and the reference non-normalized dataset for T2w-FLAIR significant features in cohort C2 discretized with bin counts of 32.

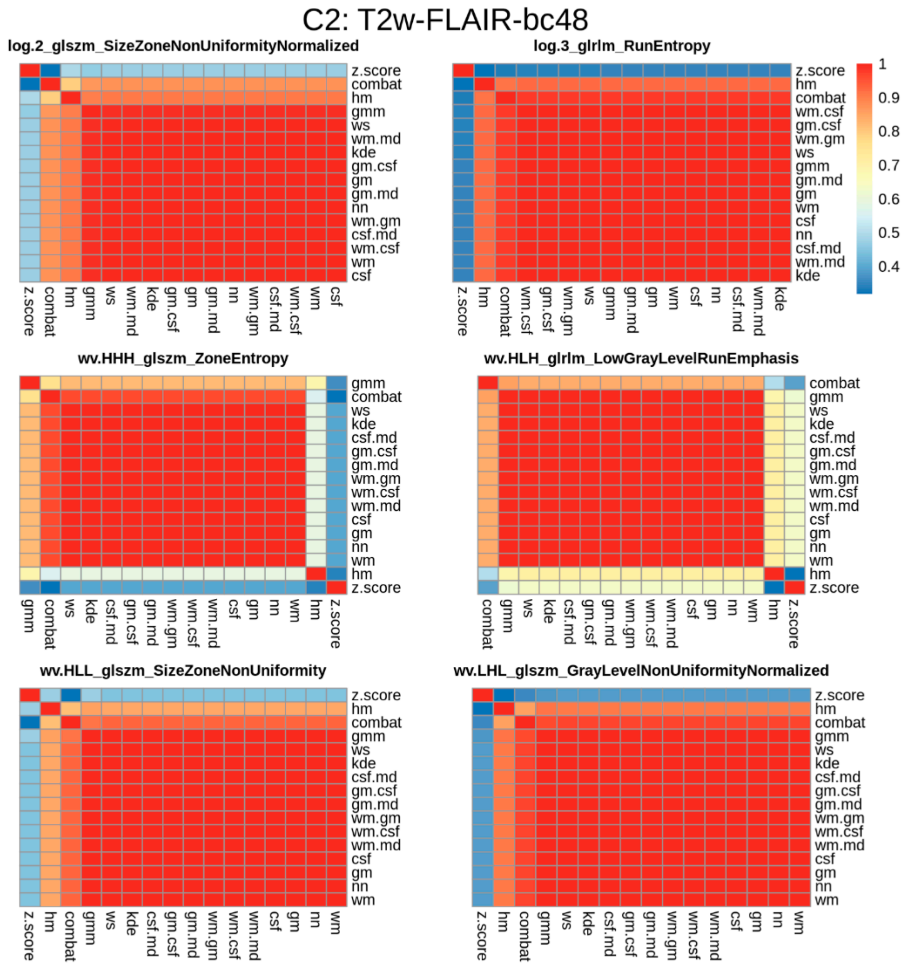

Supplementary Figure S1-38. Correlation heatmaps between the 15 different normalization methods considered and the reference non-normalized dataset for T2w-FLAIR significant features in cohort C2 discretized with bin counts of 48.

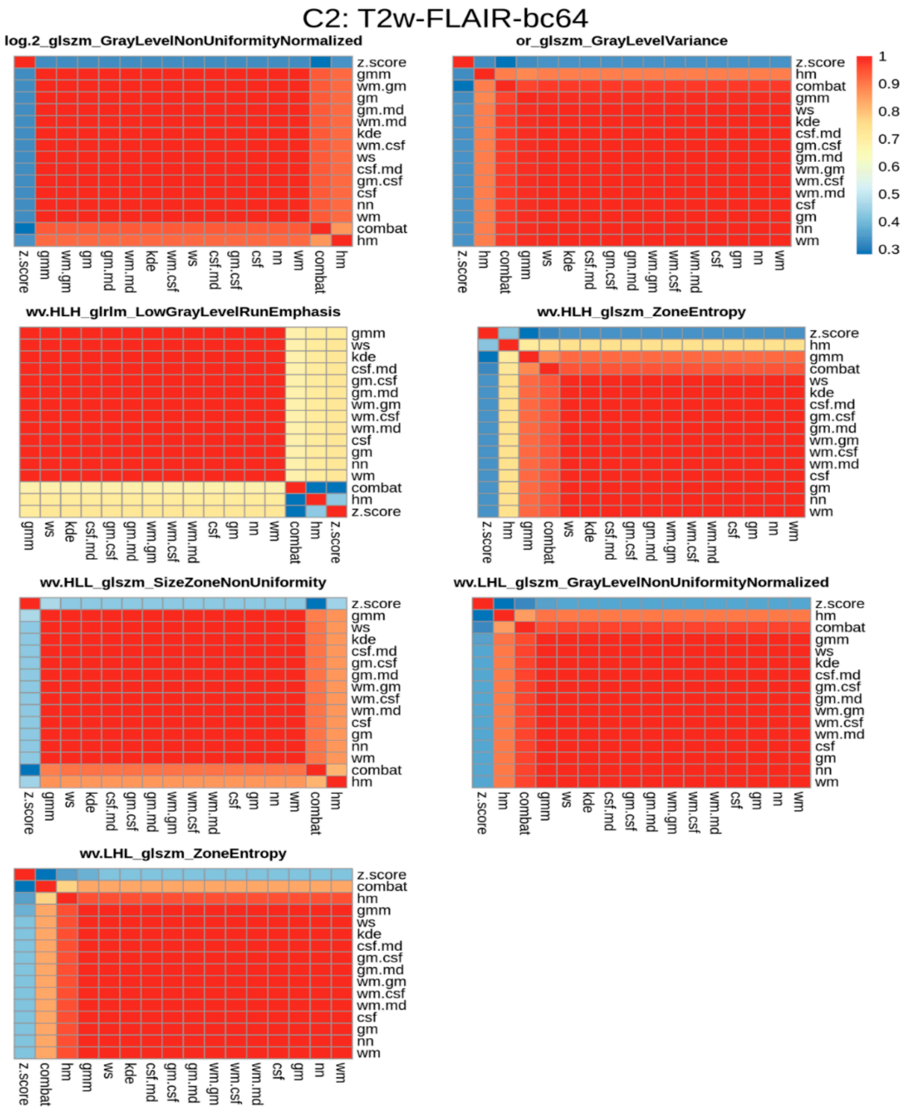

Supplementary Figure S1-39. Correlation heatmaps between the 15 different normalization methods considered and the reference non-normalized dataset for T2w-FLAIR significant features in cohort C2 discretized with bin counts of 64.

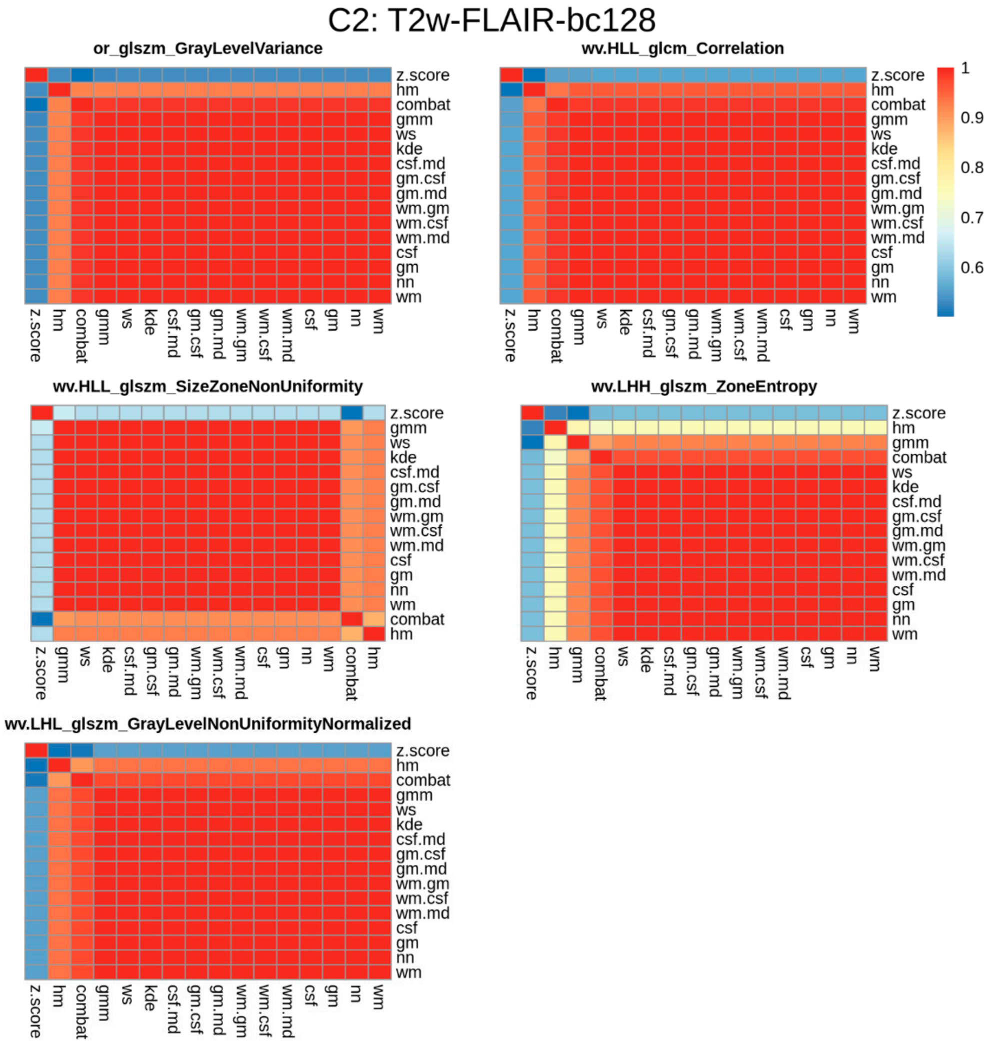

Supplementary Figure S1-40. Correlation heatmaps between the 15 different normalization methods considered and the reference non-normalized dataset for T2w-FLAIR significant features in cohort C2 discretized with bin counts of 128.
